# Cognitive and imaging markers in non-demented subjects attending a memory clinic: study design and baseline findings of the MEMENTO cohort

**DOI:** 10.1186/s13195-017-0288-0

**Published:** 2017-08-29

**Authors:** Carole Dufouil, Bruno Dubois, Bruno Vellas, Florence Pasquier, Frédéric Blanc, Jacques Hugon, Olivier Hanon, Jean-François Dartigues, Sandrine Harston, Audrey Gabelle, Mathieu Ceccaldi, Olivier Beauchet, Pierre Krolak-Salmon, Renaud David, Olivier Rouaud, Olivier Godefroy, Catherine Belin, Isabelle Rouch, Nicolas Auguste, David Wallon, Athanase Benetos, Jérémie Pariente, Marc Paccalin, Olivier Moreaud, Caroline Hommet, François Sellal, Claire Boutoleau-Bretonniére, Isabelle Jalenques, Armelle Gentric, Pierre Vandel, Chabha Azouani, Ludovic Fillon, Clara Fischer, Helen Savarieau, Gregory Operto, Hugo Bertin, Marie Chupin, Vincent Bouteloup, Marie-Odile Habert, Jean-François Mangin, Geneviève Chêne, on behalf of the MEMENTO cohort Study Group

**Affiliations:** 1 Centre Inserm U1219, Institut de Santé Publique, d’Epidémiologie et de Développement (ISPED), Bordeaux School of Public Health, Université de Bordeaux, 146 rue Léo Saignat, 33076 Bordeaux cedex, France; 2 CHU de Bordeaux, Pole de sante publique, F-33000 Bordeaux, France; 3 Institute of Memory and Alzheimer’s Disease (IM2A) and Brain and Spine Institute (ICM) UMR S 1127, Department of Neurology, AP-HP, Pitié-Salpêtrière University Hospital, Sorbonne Universities, Pierre et Marie Curie University, F-75006 Paris, France; 4 Memory Resource and Research Centre of Toulouse, CHU de Toulouse, Hôpital La Grave-Casselardit, F-31000 Toulouse, France; 5 Memory Resource and Research Centre of Lille, CHRU de Lille, Hôpital Roger Salengro, F-59000 Lille, France; 6 University Lille, INSERM U1171, F-59000 Lille, France; 7 Memory Resource and Research Centre of Strasbourg/Colmar, Department of Geriatrics, laboratoire ICube UMR 7357, FMTS, Hôpitaux Universitaires de Strasbourg, F-67000 Strasbourg, France; 8 Memory Resource and Research Centre of Paris Nord, AP-HP, Groupe Hospitalier Saint-Louis Lariboisière Fernand Widal, F-75010 Paris, France; 9 Memory Resource and Research Centre of Paris Broca, AP-HP, Hôpital Broca, F-75013 Paris, France; 10 Université Paris Descartes, Sorbonne Paris Cité, EA 4468 Paris, France; 11 Memory Resource and Research Centre of Bordeaux, CHU de Bordeaux, Hôpital Pellegrin, F-33000 Bordeaux, France; 12 Memory Resource and Research Centre of Bordeaux, CHU de Bordeaux, Hôpital Xavier Arnozan, F-33000 Bordeaux, France; 13 Memory Resource and Research Centre of Montpellier, CHU de Montpellier, Hôpital Gui de Chauliac, F-34000 Montpellier, France; 14 Memory Resource and Research Centre of Marseille, CHU de Marseille, Hôpital La Timone, F-13000 Marseille, France; 15 Memory Resource and Research Centre of Angers, CHU d’Angers, F-49000 Angers, France; 16 Memory Resource and Research Centre of Lyon, Hospices Civils de Lyon, Hôpital des Charpennes, F-69000 Lyon, France; 17 Memory Resource and Research Centre of Nice, CHU de Nice, Institut Claude Pompidou, EA 7276 CoBTeK “Cognition Behaviour Technology”, F-06100 Nice, France; 18 Memory Resource and Research Centre of Dijon, CHU Dijon Bourgogne, Hôpital du Bocage, Hôpital de Champmaillot, F-21000 Dijon, France; 19 Memory Resource and Research of Amiens, CHU Amiens Picardie, Hôpital Nord, F-80000 Amiens, France; 20 Memory Clinic, Hôpital Avicenne, AP-HP, Hôpitaux Universitaires Paris-Seine-Saint-Denis, F-93009 Bobigny, France; 21 Memory Resource and Research Centre of Saint-Etienne, CHU de Saint-Etienne, Hôpital Nord, F-42000 Saint-Etienne, France; 22 Memory Resource and Research Centre of Saint-Etienne, CHU de Saint-Etienne, Hôpital de la Charité, F-42000 Saint-Etienne, France; 23 Memory Resource and Research Centre of Rouen, Neurology Department, Rouen University Hospital, F-76031 Rouen, France; 24 Memory Resource and Research Centre of Nancy, CHU de Nancy, F-54000 Nancy, France; 25 Memory Resource and Research Centre of Toulouse, CHU de Toulouse, Hôpital Purpan, F-31000 Toulouse, France; 26 Memory Resource and Research Centre of Poitiers, CHU de Poitiers, Hôpital de La Milétrie, F-86000 Poitiers, France; 27 Memory Resource and Research Centre of Grenoble, CHU de Grenoble Alpes, Hôpital de la Tronche, F-38000 Grenoble, France; 28 Memory Resource and Research Centre of Center Region, CHRU de Tours, Hôpital Bretonneau, F-37000 Tours, France; 29 Memory Resource and Research Centre of Strasbourg/Colmar, Hôpitaux Civils de Colmar, F-68000 Colmar, France; 30 Inserm U-118, Strasbourg University, F-67000 Strasbourg, France; 31 Memory Resource and Research Centre of Nantes, CHU de Nantes, F-44000 Nantes, France; 32 Memory Resource and Research Centre of Clermont-Ferrand, CHU de Clermont-Ferrand, F-63000 Clermont-Ferrand, France; 33 Memory Resource and Research Centre of Brest, CHRU de Brest, F-29000 Brest, France; 34 Memory Resource and Research Centre of Besançon, CHU de Besançon, Hôpital Jean Minjoz, Hôpital Saint-Jacques, F-25000 Besançon, France; 35 Centre pour l’Acquisition et le Traitement des Images, NeuroSpin, I2BM, Commissariat à l’Energie Atomique, F-91400 Saclay, France; 36 Sorbonne Universités, UPMC Université Paris 06, Inserm, CNRS, Institut du cerveau et la moelle (ICM) - Hôpital Pitié-Salpêtrière, Boulevard de l’hôpital, F-75013 Paris, France; 37 Nuclear Medicine Department, Pitié-Salpêtrière University Hospital, AP-HP, F-75006 Paris, France; 38 Laboratoire d’Imagerie Biomédicale, Sorbonne Universités, UPMC Univ Paris 06, Inserm U 1146, CNRS UMR 7371, F-75006 Paris, France; 39 NeuroSpin, I2BM, Commissariat à l’Energie Atomique, Université Paris-Saclay, F-91400 Saclay, France

**Keywords:** Alzheimer’s disease, Cognitive aging, Cohort studies, Natural history studies (prognosis), Neuroimaging

## Abstract

**Background:**

The natural history and disease mechanisms of Alzheimer’s disease and related disorders (ADRD) are still poorly understood. Very few resources are available to scrutinise patients as early as needed and to use integrative approaches combining standardised, repeated clinical investigations and cutting-edge biomarker measurements.

**Methods:**

In the nationwide French MEMENTO cohort study, participants were recruited in memory clinics and screened for either isolated subjective cognitive complaints (SCCs) or mild cognitive impairment (MCI; defined as test performance 1.5 SD below age, sex and education-level norms) while not demented (Clinical Dementia Rating [CDR] <1). Baseline data collection included neurological and physical examinations as well as extensive neuropsychological testing. To be included in the MEMENTO cohort, participants had to agree to undergo both brain magnetic resonance imaging (MRI) and blood sampling. Cerebral ^18^F-fluorodeoxyglucose positon emission tomography and lumbar puncture were optional. Automated analyses of cerebral MRI included assessments of volumes of whole-brain, hippocampal and white matter lesions.

**Results:**

The 2323 participants, recruited from April 2011 to June 2014, were aged 71 years, on average (SD 8.7), and 62% were women. CDR was 0 in 40% of participants, and 30% carried at least one apolipoprotein E ε4 allele. We observed that more than half (52%) of participants had amnestic mild cognitive impairment (17% single-domain aMCI), 32% had non-amnestic mild cognitive impairment (16.9% single-domain naMCI) and 16% had isolated SCCs. Multivariable analyses of neuroimaging markers associations with cognitive categories showed that participants with aMCI had worse levels of imaging biomarkers than the others, whereas participants with naMCI had markers at intermediate levels between SCC and aMCI. The burden of white matter lesions tended to be larger in participants with aMCI. Independently of CDR, all neuroimaging and neuropsychological markers worsened with age, whereas differences were not consistent according to sex.

**Conclusions:**

MEMENTO is a large cohort with extensive clinical, neuropsychological and neuroimaging data and represents a platform for studying the natural history of ADRD in a large group of participants with different subtypes of MCI (amnestic or not amnestic) or isolated SCCs.

**Trial registration:**

Clinicaltrials.gov, NCT01926249. Registered on 16 August 2013.

**Electronic supplementary material:**

The online version of this article (doi:10.1186/s13195-017-0288-0) contains supplementary material, which is available to authorized users.

## Background

Maintaining brain health is a challenge for ageing societies as the burden of late-life brain disorder is expected to increase exponentially in the coming years [1]. Early diagnosis and intervention are therefore a priority target to defeat late-onset Alzheimer’s disease and related disorders (ADRD); however, the causes remain unknown, and no curative treatment is therefore available. Despite continuous research and recent huge progress in the identification of new biomarkers or new genes associated with brain disorders, including ADRD [2–4], the disease’s natural history, the surrogate markers of dementia and the correlates of healthy brain ageing remain largely unknown. Therefore, we are not able to fully explain the discrepancies between observations at the brain level (through neuropathological or brain imaging features) and observations at the clinical level (mainly through neuropsychological performance) [5]. This uncertainty is well illustrated by recent results of clinical trials which were successful in stopping amyloid accumulation (thought to initiate Alzheimer’s disease [AD] pathology by destroying synapses) but had no significant impact on the clinical course of ADRD [6, 7]. Improving knowledge on the natural history of ADRD involves follow-up of individuals starting from early symptoms, compatible with further progress to ADRD, until clinical dementia with an integrative phenotyping approach that combines standardised, repeated clinical investigations and cutting-edge biomarkers measurements [8, 9].

The MEMENTO cohort is a large, clinic-based cohort of participants consulting in French memory clinics and presenting with either isolated cognitive complaints or recently diagnosed mild cognitive impairment (MCI). Investigations include regular standardised clinical, brain neuroimaging and biological workup from cohort inception. The aim of MEMENTO investigators is to improve the understanding of ADRD’s natural history and identify new phenotypes of participants who will develop dementia over time.

## Methods

### Study design

**Fig. 1:**
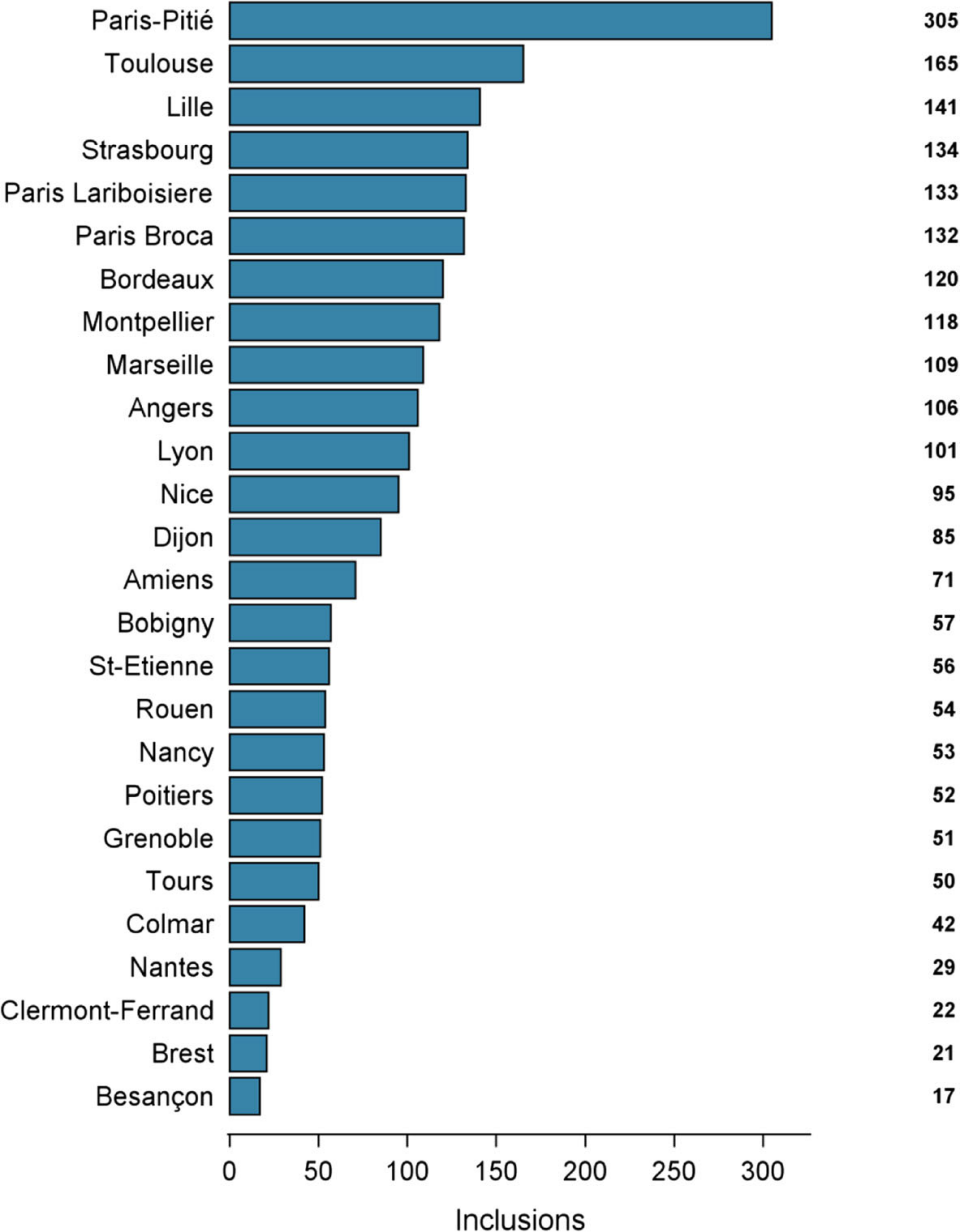
Number of included subjects by centre in the MEMENTO cohort

The MEMENTO cohort is a clinic-based study of patients presenting with a large variety of cognitive symptoms and subjective cognitive complaints (SCCs) that will be followed over a 5-year period. Between April 2011 and June 2014, among the 2449 participants screened as meeting the inclusion criteria, 2323 patients consented to participate in the study. The recruitment took place within the French national network of university-based memory clinics (Centres de Mémoires de Ressources et de Recherche [CMRR]). The 28 CMRRs comprising the network were approached because their clinical research centres had (1) the potential to include a substantial number of participants, (2) access to neuroimaging (1.5- or 3-T magnetic resonance imaging [MRI]) and (3) biobank facilities. Twenty-six CMRRs agreed to participate. The number of included subjects per CMRR ranged from 17 to 305, and Fig. 1 shows the distribution of inclusions numbers by centre.

### Selection criteria

The participants were screened for either very mild to mild cognitive impairment or isolated cognitive complaints, and they were recruited consecutively. *Very mild* to *mild cognitive impairment* was defined as (1) performing 1 SD worse than the subject’s own age, sex and education-level group mean in one or more cognitive domains, this deviation being identified for the first time through cognitive tests performed recently (less than 6 months preceding screening phase), and (2) having a Clinical Dementia Rating (CDR) [10] ≤0.5 and not being demented. The neuropsychological tests battery and the references for age, sex and education-level norms used for each test are detailed in Additional file 1. A participant was eligible for inclusion in the isolated SCCs stratum if he or she had SCCs (assessed through visual analogue scales) without any of objective cognitive deficit as defined above and was aged 60 years or older.

Exclusion criteria were as follows: being under guardianship; residence in skilled nursing facility; pregnant or breastfeeding women; AD known as being caused by gene mutations; history of intracranial surgery; neurological disease such as treated epilepsy, treated Parkinson’s disease, Huntington’s disease, brain tumour, subdural haematoma, progressive supranuclear palsy, or history of head trauma followed by persistent neurological deficits; stroke diagnosed in the last 3 months preceding enrolment visit; history of stroke followed by persistent neurological deficits; schizophrenia history (*Diagnostic and Statistical Manual of Mental Disorders, Fourth Edition* [DSM-IV], criteria); and illiteracy (unable to count or to read). Eligible adult participants had to undergo at baseline all clinical examinations, brain MRI and blood sampling. They had to have visual and auditory acuity adequate for neuropsychological testing and to have health insurance.

### Study examinations

At memory clinics, baseline data collection included socio-demographic characteristics; personal and familial medical history; neurological and physical examination (including anthropometric measurements and three measures of blood pressure after 2 minutes of rest in sitting position using the Omron M6 monitor, OMRON Healthcare, Kyoto, Japan); current medication intake with detailed recording of doses as well as treatment onset date; cognitive and non-cognitive subjective complaints (domains assessed were memory, attention, language, physical health, mood, sensory organs, general health) measured using a visual analogue scale ranging from 0 to 10; a full neuropsychological test, which consisted of the Mini Mental State Examination (MMSE) [11], digit span (forward and backward) [12], Free and Cued Selective Reminding Test [13], Delayed Matching to Sample 48 (DMS48) [14], Verbal Fluency (animals and letter p) [15], Image Naming [16], praxis assessment [17], Rey-Osterrieth Complex Figure Test [18], Trail Making Test (TMT) A and B [19, 20], Frontal Assessment Battery (FAB) [21, 22] and Clinical Dementia Rating that was completed with an informant in person if he or she was accompanying the participant, by phone otherwise; rating of neuropsychiatric symptom presence and severity using the Neuropsychiatric Inventory–Clinician (NPI-C) [23]; lifestyle indicators such as self-report of current and past alcohol consumption and smoking habits, physical activity using International Physical Activity Questionnaire [24] and leisure activity frequency; autonomy using the Instrumental Activities in Daily Living Scale and Activities of Daily Living Scale [25, 26]; and motricity using the Short Physical Performance Battery (SPPB) [27]. For CDR, NPI-C and quoting of neuropsychological tests, training sessions were organised to optimise standardisation across centres. All data were recorded via secured online electronic data capture.

The percentages of missing data entered in the electronic case report forms ranged from 0.09% to 2.12% across centres. Twenty centres had less than 1% of missing data. These numbers illustrate the high compliance rate of the centres with the MEMENTO study, as well as the efficiency of data-monitoring procedures (both on-site and centralised).

### Neuroimaging procedures

As part of the inclusion criteria, participants had to agree to undergo brain MRI, and 86% of participants had a 3.0-T MRI scan (1.5 T otherwise). ^18^F-fluorodeoxyglucose positon emission tomography (FDG-PET) was optional and was performed in 60% of participants. All neuroimaging acquisition was coordinated by the Center for Automated Treatment of Images (CATI; cati-neuroimaging.com), a platform dedicated to multicentre neuroimaging [28]. For the MEMENTO study, CATI harmonised MRI and PET imaging across a network of facilities associated with the network of French memory centres. Physicists and engineers standardised acquisitions according to a systematic qualification procedure, ensuring parameter uniformity and image quality. They had frequent contact with each acquisition site to track any hardware or software upgrades. Detailed information on scanner manufacturers and acquisition parameters for each study’s site can be found on the CATI website.

### MRI procedure

The MRI protocol harmonised by CATI is made up of the sequences described in Table 1. It guarantees compatibility with the Alzheimer's Disease Neuroimaging Initiative (ADNI) protocol to a large extent. The order of the MRI sequences corresponds to priority. Diffusion magnetic resonance imaging (dMRI) scan acquisition leads to a final sequence of 15–60 homogeneous directions according to the comfort and compliance of the subject. Non-clinical acquisitions (functional MRI, dMRI) can be considered as options to be triggered according to the compliance of the subject. Acquisition parameters were tuned for each scanner to minimise inter-site variability.

**Table 1:** Description of magnetic resonance imaging sequences in the MEMENTO cohort

Sequence number	Sequence labelling	Sequence approximate duration, minutes:seconds	Sequence compatibility with ADNI MRI protocol
1	Localizer	0:10	
2	3D T1-weighted	9:00	Yes
3	2D T2-weighted FLAIR	4:00	Yes
4	2D T2-weighted (GRE)	5:30	Yes
5	2D T2-weighted TSE/FSE single-echo	1:45	
6^a^	Resting-state fMRI BOLD EPI	10:00	
7^a^	Diffusion-weighted imaging (DTI − DWI EPI) + B0 field map	4:30 × 2–4 1:45	

*Abbreviations: ADNI* Alzheimer’s Disease Neuroimaging Initiative, *BOLD* Blood oxygen level-dependent imaging, *DTI* Diffusion tensor imaging, *DWI* Diffusion-weighted imaging, *EPI* Echo planar imaging, *FLAIR* Fluid attenuation inversion recovery, *fMRI* Functional magnetic resonance imaging, *FSE* Fast spin echo imaging, *GRE* Gradient echo imaging, *MRI* Magnetic resonance imaging, *TSE* Turbo spin echo imaging

^a^Sequences 6 and 7 were optional

### PET procedure

PET centres participating in the MEMENTO study were equipped with systems set-up between 2003 and 2014 and consisted of 10 GE Healthcare, 6 Philips and 12 Siemens Healthcare systems, as well as 15 different models of scanners. No brain-dedicated high-resolution system or 2D tomography systems were included. Therefore, we chose to harmonise patient data acquisition using phantom experiments prior to patient studies to ensure good-quality imaging data among centres [29]. Our objective was to optimise contrast recovery while preserving, if possible, spatial resolution. Two phantom studies were acquired for the qualification process of the 22 centres that agreed to perform FDG-PET: the Jaszczak phantom, composed of cold rods and six hollow spheres, and the Hoffman 3D brain phantom. Standardised uptake value measurements were also checked to assess the cross-calibration between the PET scanner and the dose calibration system. Computed tomographic (CT) image acquisition parameters were set such that the effective dose would be low (≤0.3 mSv), in accordance with the recommendations of the European Association of Nuclear Medicine [30]. Different reconstruction parameters were compared on the basis of recovery coefficients (RCs) computed for each sphere and spatial resolution estimated from the cold rods. RCs were calculated from measurements in volumes of interest (VOIs) defined on each sphere and on the background. In-house software was developed for this purpose. We also computed the image spatial resolution (ISR) using a method developed by Prieto et al. [31] to estimate the full width at half maximum of each PET scanner from the three largest hot spheres. Hoffman 3D brain phantom scans were segmented in VOIs to compute right-to-left and anterior-to-posterior cortical ratios, as well as caudate-to-white matter, putamen-to-white matter and grey matter-to-white matter ratios. For each centre, the set of optimal reconstruction parameters was chosen as the one maximising ISR and the RC without a noticeable decrease in signal-to-noise ratio.

After the set-up visit, we provided recommendations to each centre for acquisition and reconstruction parameters to be used for the MEMENTO cohort. In addition, centres were qualified after the analysis of the images of a first test patient. They received a technical manual describing in detail the procedures pertaining to patient preparation, injected dose, PET-CT image acquisition and data transfer to CATI.

Brain FDG-PET scans were obtained 30 minutes after injection of 2 MBq/kg of 2-deoxy-2-^18^F-fluoro-d-glucose. All acquisitions consisted of 3 × 5-minute frames. Images were then reconstructed using and iterative algorithm, and last, frames were realigned, averaged and quality-checked by the CATI team.

### Image analysis

The data flow between the acquisition network and the CATI centralised analysis team relied on a secured web service. A team of clinical research assistants performed quality control of the incoming data in the days following the data transfer to provide rapid feedback when required. Quality control relied on a dedicated software programme supporting the check for protocol consistency (e.g., scanner type, software version, reception coil, sequences acquired, order of sequences, sequence parameters, reconstruction parameters) and the generation of a documented series of indices characterising, for instance, acquisition slab positioning; movement; spikes and other artifacts and their localisation; and overall quality of the image through contrast, noise or intensity non-uniformity.

Validated data were moved to a centralised database at the disposal of several teams in charge of analysis. Each analysis of CATI’s portfolio was performed following a systematic procedure providing quality control indices. Below is a summary of the measurements included in MEMENTO database:

- Whole-brain and grey/white volumetry performed using the method “segment” in SPM8 software
- Hippocampal volumetry performed with SACHA software [32, 33] complemented by visual assessment done centrally at CATI by two trained doctors using the Scheltens scale [34]
- Cortical thickness computed with FreeSurfer software for each region of interest (ROI) of the Desikan-Killiany Atlas [35, 36]
- Sulcal span computed with the Morphologist method of the BrainVISA software package for each sulcus of the BrainVISA Sulci atlas [37, 38]
- White matter hyperintensity volumetry using WHASA software [39] complemented by visual assessment done centrally at CATI by two trained doctors using the Fazekas and Schmidt scale [40]
- Fractional anisotropy and mean diffusivity in large white matter bundles computed using Connectomist software [41, 42]
- Integrity of the default mode and of the salience network computed as the mean correlation across a set of Talairach coordinates
- Mean FDG-PET uptake for the ROIs of the Automated Anatomical Labeling atlas relative to the pons reference region [43], including partial volume correction
- Mean FDG-PET uptake for a set of disease-specific ROIs inferred from the ADNI database [44]

A selection of these measurements is described in this paper.

### Blood sampling

From baseline blood intake, standard biological measurements (including glycaemia, triglycerides, high-density lipoprotein, and low-density lipoprotein) were performed at local biochemistry departments. Study-specific blood sampling included serum (12 tubes of 0.25 ml), plasma ethylenediaminetetraacetic acid (EDTA; 8 tubes of 0.25 ml), total blood heparin (2 tubes of 1 ml), plasma heparin (4 tubes of 500 μg), blood EDTA without plasma (1 tube of 0.25 ml), blood heparin without plasma (1 tubes of 3 ml) and Tempus (2 tubes of 3 ml). Samples were stored in a centralised biobank (Genomic Analysis Laboratory-Biological Resource Centre [LAG-CRB], Pasteur Institut Lille, BB-0033-00071).

LAG-CRB extracted genomic DNA from peripheral blood samples using Gentra Puregene blood kits (QIAGEN, Hilden, Germany). Apolipoprotein E (APOE) genotypes were determined by KBiosciences (Hoddesdon, UK; www.kbioscience.co.uk), using their own system of fluorescence-based competitive allele-specific polymerase chain reaction. Two APOE single-nucleotide polymorphisms, rs429358 and rs7412, allowed identification of the three major APOE alleles (ε2, ε3 and ε4).

### Cerebrospinal fluid sampling

Lumbar puncture was optional and was performed in 17% of participants at baseline. Cerebrospinal fluid (CSF) was collected in polypropylene tubes following standardised conditions and using an atraumatic needle. Each CSF sample was transferred to the CSF bank within 4 h after collection and was centrifuged at 1000 × *g* at 4 °C for 10 minutes. CSF samples were aliquoted in polypropylene tubes (16 tubes of 250 μl) and stored at −80 °C. All tubes were shipped for storage in a centralised biobank (LAG-CRB, Pasteur Institut Lille, BB-0033-00071). Measurements of CSF amyloid-β 42 peptide (Aβ_42_), CSF Aβ_40_, total tau, and phosphorylated tau (p-tau181) levels are ongoing using the standardised commercially available INNOTEST sandwich enzyme-linked immunosorbent assay (Fujirebio, Ghent, Belgium).

### Follow-up

Longitudinal follow-up took place every 6 months. Table 2 describes the schedule of overall evaluations. During follow-up, all incident cases of dementia (DSM-IV criteria for dementia and National Institute of Neurological and Communicative Disorders and Stroke/Alzheimer’s Disease and Related Disorders Association criteria for AD) [45, 46] were reviewed by an independent committee.

**Table 2:** Schedule of evaluation in the MEMENTO cohort over 60 months of follow-up

	Schedules of evaluation by follow-up wave (months)										
	Baseline	M6	M12	M18	M24	M30	M36	M42	M48	M54	M60
Socio-demographic characteristics	■	□	■	□	■	□	■	□	■	□	■
Medical history or incident events	■	□	■	□	■	□	■	□	■	□	■
Physical, neurological examinations	■	□	■	□	■	□	■	□	■	□	■
Medication	■	□	■	□	■	□	■	□	■	□	■
Clinical Dementia Rating	■	■	■	■	■	■	■	■	■	■	■
Full neuropsychological battery	■	■	■	■	■	■	■	■	■	■	■
Subjective complaints	■	■	■	■	■	■	■	■	■	■	■
Neuropsychiatric Inventory	■	■	■	■	■	■	■	■	■	■	■
Lifestyle	■	■	■	■	■	■	■	■	■	■	■
Autonomy in activities of daily living	■	■	■	■	■	■	■	■	■	■	■
Motricity (SPPB)	■	■	■	■	■	■	■	■	■	■	■
Quality of life (EQ-5D)	■	■	■	■	■	■	■	■	■	■	■
Social sciences and health economic questionnaires	■	■	■	■	■	■	■	■	■	■	■
Blood sampling laboratory assessment	■		■		■		■		■		■
Biobank	■				■				■		
DNA sample collection	■				■				■		
RNA collection	■				■				■		
Brain structural MRI	■				■				■		
^18^F-FDG-PET scan	☑				☑				☑		
Lumbar puncture	☑				☑				☑		

*Abbreviations: FDG-PET* ^18^F-fluorodeoxyglucose positon emission tomography, *MRI* Magnetic resonance imaging, *SPPB* Short Physical Performance Battery

■ At examination centre

□ By phone or at examination centre

☑ Optional, at examination centre

### Statistical analysis

Baseline characteristics of participants were summarised by cognitive categories (isolated SCC or MCI staging according to Petersen criteria [47]; i.e., single-domain amnestic mild cognitive impairment [aMCI], multi-domain aMCI, single-domain non-amnestic mild cognitive impairment [naMCI], multi-domain naMCI), age categories in years (≤ 60; 60 – 69, 70 – 79, ≥ 80), sex and APOE genotype. An isolated SCC category comprised participants without impairment at any of the cognitive tests of the screening battery (impairment defined as a score ≥1.5 SD worse than a participant’s own age, sex or education-level group mean in a cognitive domain).

Definitions used were as follows: for highest diploma, at least baccalaureate degree (yes/no); for cardiovascular burden, diabetes (self-reported diabetes or anti-diabetic drug intake of glycaemia > 7 mmol/L), hypertension (anti-hypertensive drug intake or mean of three blood pressure measurements either ≥ 140 mmHg for systolic blood pressure or ≥ 90 mmHg for diastolic blood pressure), dyslipidaemia (plasma cholesterol > 6.24 mmol/L or use of any lipid-lowering drugs), history of cardiovascular disease (self-reported history of myocardial infarction, surgical bypass, stroke, peripheral artery disease, angina pectoris); for neuropsychiatric symptoms, apathy, depression, anxiety based on NPI-C; for physical impairment and motricity, number of limitations in instrumental activities of daily living (none, 1, 2 or more) and SPPB score; for genetic AD risk, number of copies of the APOE ε4 allele (0, 1 or 2); for cognitive level, performance on all cognitive tests; for brain MRI biomarkers, hippocampal volume (by hemisphere), brain parenchymal fraction (grey matter + white matter volumes divided by total intracranial volume), total white matter lesion (WML) volume and mean cortical thickness by hemisphere; and for FDG-PET biomarkers, mean FDG uptake values normalised to the pons in five AD-specific regions derived from the ADNI cohort (angular and parietal inferior right, parietal inferior left, precuneus and cingulum posterior left, temporal inferior left, temporal inferior right) [48].

Comparisons across categories are presented with percentages when baseline characteristics are categorical and as means with SDs when baseline characteristics are continuous. *P* values were derived from multivariable models adjusting for centre, age, sex, education level and CDR (0 vs. 0.5) (logistic or multinomial regression for categorical baseline characteristics, generalised linear models for continuous baseline characteristics). In multivariable analyses of hippocampal volume, WML volume and cortical thickness, models were additionally adjusted for total intracranial volume as a potential confounding factor.

Analyses were performed using SAS version 9.3 software (SAS Institute, Cary, NC, USA) and Stata release 14 software (StataCorp LP, College Station, TX, USA). Results are reported following the Methods in Longitudinal Research on Dementia guidelines [49].

## Results

Table 3 presents the baseline characteristics of the MEMENTO participants. Sixty-two percent were women; mean age at inclusion was 70.9 years (SD 8.7); and almost 10% were 60 years old or younger (Table 3). About half of the participants had reached a baccalaureate degree education level, more frequently among men (59.8%) than among women (51.8%). The most frequent cardiovascular risk factor was hypertension (33.4%). Anxiety was reported for 42% of participants. Thirteen percent of participants had at least one limitation in instrumental activities of daily living. Thirty percent of participants carried at least one copy of the APOE ε4 allele (3.4% were APOEε4/ε4). The mean MMSE score was 27.9 (SD 1.9). The mean right hippocampal volume was 2.76 cm^3^.

**Table 3:** Baseline characteristics of the MEMENTO cohort

	Total
No. of subjects	2323
Female sex, %	61.8
Age in years, mean (SD)	70.9 (8.7)
Baccalaureate degree or higher education level, %	54.8
Diabetes, %	7.4
Hypertension, %	33.8
Dyslipidaemia, %	27.8
History of cardiovascular disease, %	11.8
Apathy, %	17.2
Depression,%	33.6
Anxiety, %	41.8
No. of limitations in IADL, %	
One	10.3
Two or more	2.6
SPPB score, mean (SD)	10.6 (1.9)
At least one APOE ε4 allele for carried, %	30.0
CDR Sum of Boxes, mean (SD)	0.6 (0.7)
MMSE score, mean (SD)	27.9 (1.9)
Verbal fluency, letter P, mean (SD)	20.4 (7.2)
Verbal fluency, animals, mean (SD)	28.3 (8.8)
DMS48, immediate recall, mean (SD)	44.7 (4.0)
Praxis total score, mean (SD)	21.8 (1.6)
TMT A, time in seconds, mean (SD)	2.1 (0.93)
TMT B, time in seconds, mean (SD)	5.2 (4.4)
FCSRT total immediate free recall, mean (SD)	25.9 (8.4)
FCSRT total free and cued delayed recall, mean (SD)	14.9 (2.3)
Digit span standardised score, mean (SD)	9.9 (3.1)
Rey Complex Figure Test, immediate copy score, mean (SD)	32.9 (4.3)
Rey Complex Figure Test, 3-minute copy score, mean (SD)	15.1 (7.0)
FAB score, mean (SD)	16.2 (1.9)
DO 80 score, mean (SD)	78.6 (3.3)
Hippocampal volume, right, cm^3^, mean (SD)	2.76 (0.42)
Hippocampal volume, left, cm^3^, mean (SD)	2.66 (0.41)
Brain parenchymal fraction, %, mean (SD)	81.5 (1.3)
White matter lesion volume, cm^3^, mean (SD)	10.3 (13.8)
Cortical thickness, right, mm, mean (SD)	2.32 (0.11)
Cortical thickness left, mm, mean (SD)	2.33 (0.11)
Angular and parietal inferior, right, FDG uptake, mean (SD)	1.74 (0.21)
Parietal inferior, left, FDG uptake, mean (SD)	1.71 (0.21)
Precuneus and cingulum, posterior left, FDG uptake, mean (SD)	1.94 (0.24)
Temporal, inferior left, FDG uptake, mean (SD)	1.62 (0.17)
Temporal, inferior right, FDG uptake, mean (SD)	1.64 (0.17)

*Abbreviations: APOE* Apolipoprotein E, *CDR* Clinical Dementia Rating, *DO 80* Dénomination Orale D’images, *DMS48* Delayed Matching to Sample 48, *FAB* Frontal Assessment Battery, *FCSRT* Free and Cued Selective Reminding Test, *FDG* ^18^F-fluorodeoxyglucose, *IADL* Instrumental Activities of Daily Living, *MMSE* Mini Mental State Examination, *SPPB* Short Physical Performance Battery, *TMT* Trail Making Test

Table 4 presents the distribution of baseline characteristics in the five defined cognitive categories. Sixteen percent of participants (*n* = 370) had isolated SCCs. Two centres (Nantes and Brest) included no participant in that stratum, whereas the Paris Pitié-Salpêtrière University Hospital centre had the largest proportion of SCC participants (23.4%) because it ran a sub-study focused on this phenotype (the INSIGHT study, http://icm-institute.org/en/alzheimer-en/). In the SCC group, the cognitive domain in which SCCs were the highest was memory (mean 4.1, SD 2.7), and it was the lowest for language (mean 3.2, SD 2.4).

**Table 4:** Association between baseline characteristics and cognitive categories in the MEMENTO cohort

	Cognitive categories					
	Isolated cognitive complaint	Single-domain aMCI	Multi-domain aMCI	Single-domain naMCI	Multi-domain naMCI	*P* value^a^
No. of subjects	370	207	998	392	346	
Female sex, %	65.4	51.2	57.3	66.8	71.7	<0.0001
Age in years, mean (SD)	69.8 (8.0)	68.8 (9.6)	70.9 (9.0)	71.6 (8.0)	72.2 (8.6)	<0.0001
Baccalaureate degree or above, %	65.8	58.0	47.3	61.9	55.2	<0.0001
Diabetes, %	4.9	7.3	8.9	5.6	7.8	0.07
Hypertension, %	28.4	34.3	35.4	31.6	37.0	0.08
Dyslipidaemia, %	26.0	26.1	30.5	26.3	25.1	0.19
History of cardiovascular disease, %	9.0	10.7	13.5	11.0	11.9	0.21
Apathy, %	7.1	15.7	22.2	14.3	17.7	<0.0001
Depression, %	27.3	31.1	38.9	27.6	33.4	<0.0001
Anxiety, %	32.1	43.3	46.9	36.9	42.1	<0.0001
Number of limitations in IADL, %						
One	8.3	7.4	10.1	11.2	13.4	0.04
Two or more	1.7	2.6	3.3	0.8	3.3	
SPPB score, mean (SD)	10.9 (0.09)	10.5 (0.13)	10.3 (0.06)	10.7 (0.09)	10.5 (0.10)	<0.0001
At least one APOE ε4 allele carried, %	26.0	27.3	33.5	20.9	26.0	0.007
CDR Sum of Boxes, mean (SE)	0.33 (0.03)	0.50 (0.05)	0.85 (0.02)	0.38 (0.03)	0.53 (0.04)	<0.0001
MMSE score, mean (SE)	28.7 (0.09)	28.3 (0.12)	27.3 (0.06)	28.5 (0.09)	27.9 (0.10)	<0.0001
Verbal fluency, letter P, mean (SE)	24.0 (0.34)	22.9 (0.45)	18.5 (0.21)	22.0 (0.33)	18.7 (0.35)	<0.0001
Verbal fluency, animals, mean (SE)	33.7 (0.41)	31.6 (0.55)	25.0 (0.25)	21.3 (0.40)	26.9 (0.41)	<0.0001
DMS48, immediate recall, mean (SE)	46.9 (0.18)	43.9 (0.24)	42.5 (0.11)	46.9 (0.17)	46.8 (0.19)	<0.0001
Praxis total score, mean (SE)	22.7 (0.08)	22.6 (0.10)	21.5 (0.05)	22.1 (0.07)	21.2 (0.08)	<0.0001
TMT A time, seconds, mean(SE)	1.6 (0.04)	1.6 (0.06)	2.3 (0.03)	1.9 (0.4)	2.3 (0.05)	<0.0001
TMT B time, seconds, mean (SE)	3.5 (0.21)	3.7 (0.29)	6.4 (0.13)	4.2 (0.21)	5.7 (0.22)	<0.0001
FCSRT, total immediate free recall, mean (SE)	30.7 (0.36)	25.7 (0.48)	21.9 (0.22)	29.8 (0.34)	28.0 (0.37)	<0.0001
FCSRT, total free and cued delayed recall, mean (SE)	15.8 (0.11)	14.8 (0.14)	13.9 (0.10)	15.8 (0.10)	15.8 (0.11)	<0.0001
Digit span, standardised score, mean (SE)	11.2 (0.15)	10.9 (0.20)	9.4 (0.09)	10.4 (0.15)	8.9 (0.16)	<0.0001
Rey Complex Figure Test, immediate copy score, mean (SE)	34.0 (0.22)	33.6 (0.29)	32.1 (0.13)	33.8 (0.21)	32.5 (0.22)	<0.0001
Rey Complex Figure Test, 3-minute copy score, mean (SE)	19.3 (0.31)	14.4 (0.42)	11.6 (0.19)	18.8 (0.30)	16.4 (0.34)	<0.0001
FAB score, mean (SE)	17.5 (0.08)	17.5 (0.11)	15.5 (0.05)	16.5 (0.08)	15.4 (0.09)	<0.0001
DO 80 score, mean (SE)	79.5 (0.17)	79.1 (0.22)	77.9 (0.11)	79.2 (0.16)	78.7 (0.17)	<0.0001
Hippocampal volume, right, cm^3^, mean (SE)	2.84 (0.02)	2.77 (0.03)	2.68 (0.01)	2.84 (0.02)	2.78 (0.02)	<0.0001
Hippocampal volume, left, cm^3^, mean (SE)	2.75 (0.02)	2.65 (0.03)	2.60 (0.01)	2.73 (0.02)	2.67 (0.02)	<0.0001
Brain parenchymal fraction, %, mean (SE)	81.6 (0.06)	81.5 (0.08)	81.4 (0.04)	81.7 (0.06)	81.4 (0.06)	<0.0001
White matter lesion volume, cm^3^, mean (SE)	7.9 (0.76)	10.5 (1.0)	12.0 (0.46)	9.3 (0.74)	9.3 (0.78)	<0.0001
Cortical thickness, right, mm, mean (SE)	2.34 (0.01)	2.33 (0.01)	2.31 (0.003)	2.34 (0.005)	2.32 (0.006)	<0.0001
Cortical thickness, left, mm, mean (SE)	2.34 (0.005)	2.33 (0.01)	2.32 (0.003)	2.34 (0.005)	2.32 (0.006)	0.0005
Angular and parietal inferior, right, FDG uptake, mean (SE)	1.79 (0.01)	1.77 (0.02)	1.70 (0.01)	1.79 (0.01)	1.74 (0.01)	<0.0001
Parietal inferior, left, FDG uptake, mean (SE)	1.75 (0.01)	1.74 (0.02)	1.67 (0.01)	1.76 (0.01)	1.71 (0.01)	<0.0001
Precuneus and cingulum, posterior left, FDG uptake, mean (SE)	2.00 (0.01)	1.97 (0.02)	1.89 (0.01)	1.99 (0.01)	1.94 (0.01)	<0.0001
Temporal, inferior left, FDG uptake, mean (SE)	1.65 (0.01)	1.64 (0.01)	1.58 (0.01)	1.65 (0.01)	1.61 (0.01)	<0.0001
Temporal, inferior right, FDG uptake, mean (SE)	1.68 (0.01)	1.65 (0.01)	1.60 (0.01)	1.66 (0.01)	1.64 (0.01)	<0.0001

*Abbreviations: aMCI* Amnestic mild cognitive impairment, *APOE* Apolipoprotein E, *CDR* Clinical Dementia Rating, *DO 80* Dénomination Orale D’images, *DMS48* Delayed Matching to Sample 48, *FAB* Frontal Assessment Battery, *FCSRT* Free and Cued Selective Reminding Test, *FDG* ^18^F-fluorodeoxyglucose, *IADL* Instrumental Activities of Daily Living, *MMSE* Mini Mental State Examination, *naMCI* Non-amnestic mild cognitive impairment, *SPPB* Short Physical Performance Battery, *TMT* Trail Making Test

^a^ *P* value adjusted for centre, age, sex and education level computed from polytomous logistic regression model for categorical variables, generalised linear model for continuous variables

More than half of participants (52.1%, *n* = 1205) had aMCI, among whom one-sixth (*n* = 207) had single-domain aMCI. About one-third of participants (31.9%, *n* = 738) had naMCI, almost fairly distributed between single-domain naMCI and multi-domain naMCI. Women were significantly more represented in the isolated SCC and naMCI groups. On average, participants were older in the naMCI and multi-domain aMCI categories than in the other categories. The proportion of participants with higher education attainment was significantly greater in the isolated SCC and single-domain naMCI groups. There were no major differences in cardiovascular risk factors across cognitive categories. Among NPI symptoms, apathy and depression were more frequent in multi-domain MCI participants (aMCI and naMCI), whereas anxiety was more frequently reported among participants with aMCI and participants with multi-domain naMCI. In multivariable analyses adjusted for age, sex, education level and total intracranial volume, all MRI biomarkers were found to be consistently more severe in those with aMCI, even for WML load (Table 4). There were also differences in mean FDG uptake between cognitive categories, with a consistent hypometabolism in participants with multi-domain aMCI. Additional file 2: Table S1 displays statistical significance data for comparisons of baseline characteristics across two-by-two cognitive categories.

Additional file 2: Table S2 shows baseline characteristics by age categories and sex. All cardiovascular risk factors except diabetes were more frequent in men than in women, as well as with increasing age. Anxiety frequency decreased with increasing age. APOE genotype was not related to age or sex. As expected, for all neuropsychological tests, mean performance decreased linearly with increasing age. As for sex-related differences, men had, on average, significantly higher performances than women at praxis, visuo-spatial abilities (Rey-Osterrieth Complex Figure Test immediate and 3-minute copies). Women had, on average, significantly higher scores than men for verbal fluency (letter P only), memory tests (DMS48 immediate recall and Free and Cued Selective Reminding Test sum of immediate total recall and sum of free and cued delayed recalls), and executive function (FAB). For all selected neuroimaging markers, the older the participant, the worse the marker level. There was, on average, a volume difference of 0.59 cm^3^ in mean right hippocampal volume between participants ≤ 60 years old and those who were ≥ 80 years old. Similar trends were observed for the left hippocampus. The mean WML load was five times lower in those < 60 years old (3.2 cm^3^, SD 5.5) than in those aged ≥ 80 years old (16.1 cm^3^, SD 16.9). Crude comparisons showed smaller average volumes of cerebral structures (hippocampus, WMLs, cortical thickness) in women, with differences being no more significant after controlling for intracranial volume. All regional markers of glucose metabolism (FDG-PET) were lower, on average, with age, and women had higher FDG uptake in all disease-specific regions.

Additional file 2: Table S3 shows the participants’ characteristics by APOE ε4 allele copy number. There was a strong positive association between dyslipidaemia and APOEε4 that was not observed for the other cardiovascular indicators. Apathy and anxiety were significantly more frequent in APOEε4/ε4 carrier participants. Regarding cognitive performance, a significant association between number of copies of APOEε4 and lower cognition was observed for MMSE and tests assessing either memory or executive function (TMT and FAB). Regarding MRI biomarkers, only hippocampal and WML volumes were significantly related to APOEε4 genotype. Mean hippocampal volumes decreased linearly with number of copies of APOEε4, whereas WML load was much larger in APOEε4/ε4 participants. For FDG-PET markers, lower uptake was observed with increasing number of copies of APOEε4 in all selected ROIs.

## Discussion

A large sample of 2323 non-demented persons recently diagnosed with cognitive deficits or isolated cognitive complaints was enrolled in the MEMENTO cohort. Participants will have longitudinal multimodal assessments of clinical features as well as biological, genetic and neuroimaging biomarkers using standardised and highly reproducible techniques [50].

While a majority of newly diagnosed participants present with memory deficits as their first symptoms, almost one-third of the cohort has exclusively non-memory deficits. The neuroimaging correlates of the different MCI subtypes suggest interesting patterns. As expected, neurodegenerative neuroimaging markers (hippocampal volume, cortical thickness and brain parenchymal fraction) tend to be lower among participants with aMCI than in those with naMCI. It is also in the participants with aMCI that WML load is the largest, suggesting interactions between vascular and neurodegenerative features [51]. In the European DESCRIPA prospective cohort [52] (*N* = 881), the proportion of participants with naMCI was estimated to be 22% in the sub-group of participants with CSF samples available, and the follow-up was 3 years, which did not allow in-depth investigation of the sequential pattern of cognitive decline trajectories in these individuals compared with participants with aMCI. The MEMENTO cohort represents a powerful resource to complement investigations on the natural history of ADRD in participants whose first symptoms are not memory-specific [53, 54].

In addition, carriers of APOEε4 tend to have lower cognitive performance on memory and executive function tests, whereas differences in MRI biomarkers are seen for hippocampal volume but not for the other global atrophy markers (brain parenchyma, cortical thickness). It is also striking to observe a much larger WML load among participants who are carriers of APOEε4/ε4, underlining once again the need for further investigation of the synergistic effect of neurodegenerative processes and small vessel diseases on future dementia risk, which will be possible in the MEMENTO cohort with follow-up data.

The preliminary results (as of May 1, 2017) in the MEMENTO cohort suggest a dementia incidence of 3.2 per 100 person-years (two-thirds of cases being AD), and 90% of participants had at least one follow-up visit. These numbers are in line with the way the study was powered [55–58]. We chose not to present findings on clinical changes yet, however, because the follow-up (median 2.7 years on May 1, 2017) is insufficient to draw conclusions. Compared with other clinical studies worldwide, the MEMENTO cohort does not focus only on memory deficits [59] as the first symptoms and offers the opportunity to study the evolution of patients with a large spectrum of cognitive deficits. In MEMENTO, 370 participants have isolated SCCs. Their baseline neuroimaging or genetic biomarkers do not suggest major differences from MCI participants, as expected; moreover, they are an interesting group to follow because they also represent the target of most recent intervention studies, such as the A4 trial [60].

The MEMENTO study is being enriched through the addition of biomarkers that might be available in the near future. Lumbar puncture is proposed to participants at each visit and repeated at least every 2 years, and the Amyging (“AMYloid imaGING”) sub-study is ongoing with an aim of enrolling a sample of 700 patients who will benefit from PET amyloid imaging (using ^18^F-florbetapir or ^18^F-flutemetamol radioligands). Participation in the sub-study was proposed to all participants at any time during their follow-up.

This cohort has potential limitations and indisputable strengths. The recruitment occurred in clinics linked to university settings to allow performance of high-quality imaging and biobanking. This might have resulted in traditional selection through reference centres, as indicated by a high proportion of highly educated individuals. Nevertheless, almost all memory clinics participated and are distributed across France. In addition, this cohort has achieved the recruitment of a number of participants allowing sufficient power for many analyses. There are discrepancies in the number of participants included by centre. The present analyses were adjusted for centre, and we also checked for potential interactions. This will be done systematically in future analyses. The MEMENTO cohort design did not aim at being representative of either the general population or the CMRR active list. However, if questions on generalisability of the findings occur in the future, we have access to two databases that will allow correction for selection: (1) the French Alzheimer Databank (Banque Nationale Alzheimer), which contains demographic, diagnostic and treatment information of persons consulting at the 26 CMRRs participating in MEMENTO; and (2) the French data of the Survey on Health, Ageing and Retirement in Europe (SHARE) (general population-representative households with at least one individual aged ≥ 50 years) that include socio-demographic and health status variables.

MEMENTO is built as a platform available to researchers. Indeed, external researchers can request access to data via collaborations with the study group through enquiry to the corresponding author.

## Conclusions

The study design of the MEMENTO cohort, as well as the variety of the data collected, is a powerful resource for the discovery and validation of disease mechanisms, as well as candidate biomarkers that are needed for earlier diagnosis of AD and identification of effective preventive or early interventions. Findings derived from the MEMENTO cohort could lead to identification of biomarkers, alone or in combination, that allow stratification of patients based on phenotypes of interest (e.g., disease subtypes, prognosis and response to future therapy).

## Additional files

Additional file 1: Description of neuropsychological battery. (DOCX 29 kb)

Additional file 2: Table S1. Statistical significance of two-by-two cognitive categories comparisons of baseline characteristics distributions: the MEMENTO cohort. **Table S2.** Baseline characteristics by age group and sex: the MEMENTO cohort. **Table S3.** Association between baseline characteristics and number of copies of ε4 allele of APOE genotype: the MEMENTO cohort. (DOCX 47 kb)

Additional file 3: Memento Study Group list. (DOCX 28 kb)

## Abbreviations

Aβ: Amyloid-β
AD: Alzheimer’s disease
ADNI: Alzheimer’s Disease Neuroimaging Initiative
ADRD: Alzheimer’s disease and related disorders
aMCI: Amnestic mild cognitive impairment
APOE: Apolipoprotein E
BOLD: Blood oxygen level-dependent imaging
CATI: Center for Automated Treatment of Images
CDR: Clinical Dementia Rating
CMRR: Centres de Mémoires de Ressources et de Recherche
CSF: Cerebrospinal fluid
CT: Computed tomographic
dMRI: Diffusion magnetic resonance imaging
DO 80: Dénomination Orale D’images
DMS48: Delayed Matching to Sample 48
DSM-IV: *Diagnostic and Statistical Manual of Mental Disorders, Fourth Edition*
DTI: Diffusion tensor imaging
DWI: Diffusion-weighted imaging
EDTA: Ethylenediaminetetraacetic acid
EPI: Echo planar imaging
FAB: Frontal Assessment Battery
FCSRT: Free and Cued Selective Reminding Test
FDG-PET: ^18^F-fluorodeoxyglucose positon emission tomography
FLAIR: Fluid attenuation inversion recovery
fMRI: Functional magnetic resonance imaging
FSE: Fast spin echo imaging
GRE: Gradient echo imaging
IADL: Instrumental Activities of Daily Living
ISR: Image spatial resolution
LAG-CRB: Genomic Analysis Laboratory-Biological Resource Centre
MCI: Mild cognitive impairment
MMSE: Mini Mental State Examination
MRI: Magnetic resonance imaging
naMCI: Non-amnestic mild cognitive impairment
NPI-C: Neuropsychiatric Inventory–Clinician
p-tau181: Phosphorylated tau
RC: Recovery coefficient
ROI: Region of interest
SCC: Subjective cognitive complaint
SPPB: Short Physical Performance Battery
TMT: Trail Making Test
TSE: Turbo spin echo imaging
VOI: Volume of interest
WML: White matter lesion
