# Supplementary material for: Cognitive and imaging markers in non-demented subjects attending a memory clinic: study design and baseline findings of the MEMENTO cohort
Source: Alzheimers Res Ther. 2017 Aug 29;9:67. doi: 10.1186/s13195-017-0288-0 (PMC5576287; doi:10.1186/s13195-017-0288-0)
Supplement: Supplementary file 1 — Description of neuropsychological battery. (DOCX 29 kb) [file 13195_2017_288_MOESM1_ESM.docx]

Additional File 1. DESCRIPTION OF NEUROPSYCHOLOGICAL BATTERY

The neuropsychological battery evaluated multiple areas of cognition as follows:

- *Global cognition*:
  - The Mini-Mental State Examination (MMSE) [1]
    The MMSE consists of a set of standardized questions and tests to assess a participant’s mental status and identifies the participant’s global level of impairment.
- *Short term memory*:
  - Digit span (forward and backward) [2]

The tests consists in repeating dictated series of digits (e.g., 4 1 7 9) forwards and other series backwards. Series begin with two digits and keep increasing in length, with two trials at each length.

- *Long term memory:*
  - Free and Cued selective reminding Test[3]

The tests gives a measure of memory under conditions that control encoding and cognitive processing in order to obtain an assessment of memory unconfounded by normal age related changes in cognition.

- - Delayed Matching to Sample 48 (DMS48)[4]

The test consists in a visual recognition memory task.

- *Language and semantic Memory*
  - Verbal Fluency [5]

The test consists in producing as many words as possible within two categories in two minutes. One category is semantic (animals), the other one is phonemic (begin with letter p).

- - Image Naming (DO 80) [6]

The test consists in a set of 80 black and white line drawings pictures presented to the participant who is asked to name them.

- *Praxis*[7]
  - The test gives an assessment of gestural ideational and ideomotor praxis. It consists in asking to participants to repeat a series of gestures with or without significance.
- *Visuo Spatial abilities[8]*

Rey-Osterrieth Complex Figure Test is a neuropsychological assessment in which examinees are asked to reproduce a complex line drawing, first by copying and then from memory at 3 minutes.

- *Attention and executive functions :*
  - Trail Making Test Part A and B[9, 10]

The test consists of 25 circles distributed over a sheet of paper. In Part A, the circles are numbered 1 – 25, and the participant should draw lines to connect the numbers in ascending order. In Part B, the circles include both numbers (1 – 13) and letters (A – L); as in Part A, the participant draws lines to connect the circles in an ascending pattern, but with the added task of alternating between the numbers and letters (i.e., 1-A-2-B-3-C, etc.). The time in seconds to complete the task is recorded.

- - Frontal assessment Battery [11, 12]

The test has been designed to assess frontal lobe functions. It consists in six subtests exploring the following: conceptualization, mental flexibility, motor programming, sensitivity to interference, inhibitory control, and environmental autonomy.

- Optional Neuropsychological Battery
  - Visuospatial span (forward and backward) [13]

The test is a subtest of the Wechsler battery that assesses spatial memory.

- - *Visuo Spatial abilities[8]*

Rey-Osterrieth Complex Figure Test copy at 30 minutes.

**REFERENCES**

1. Hugonot-Diner L: MMS version consensuelle GRECO. In La consultation en gériatrie. Edited by MASSON. Paris; 2001: 13-20

2. Wechsler D: Wechsler Memory Scale. New-York, NY: Psychological Corporation; 1975.

3. Grober E, Buschke H, Crystal H, Bang S, Dresner R: Screening for dementia by memory testing. Neurology 1988, 38:900-903.

4. Barbeau E, Didic M, Tramoni E, Felician O, Joubert S, Sontheimer A, et al: Evaluation of visual recognition memory in MCI patients. Neurology 2004, 62:1317-1322.

5. Thurstone LL: Psychophysical analysis. By L. L. Thurstone, 1927. Am J Psychol 1987, 100:587-609.

6. Deloche G, Hannequin D, Dordain M, Perrier D, Pichard B, Quint S, et al: Picture confrontation oral naming: performance differences between aphasics and normals. Brain Lang 1996, 53:105-120.

7. Peigneux P, Van der Liden M: Presentation d'une batterie neuropsychologique et cognitive pour l'evaluation de l'apraxie gestuelle. vol. 10. pp. 311-362; 2000:311-362.

8. Benton AL, Varney NR, Hamsher KD: Visuospatial judgment. A clinical test. Arch Neurol 1978, 35:364-367.

9. Reischies FM, Neu P: Comorbidity of mild cognitive disorder and depression--a neuropsychological analysis. Eur Arch Psychiatry Clin Neurosci 2000, 250:186-193.

10. Tombaugh TN: Trail Making Test A and B: normative data stratified by age and education. Arch Clin Neuropsychol 2004, 19:203-214.

11. Dubois B, Slachevsky A, Litvan I, Pillon B: The FAB: a Frontal Assessment Battery at bedside. Neurology 2000, 55:1621-1626.

12. Slachevsky A, Villalpando JM, Sarazin M, Hahn-Barma V, Pillon B, Dubois B: Frontal assessment battery and differential diagnosis of frontotemporal dementia and Alzheimer disease. Arch Neurol 2004, 61:1104-1107.

13. Wechsler D: Manual for the Wechser Adult Intelligence Scale-Revised. New York: NY: Psychological Corporation; 1981.
