# Supplementary material for: Cognitive and imaging markers in non-demented subjects attending a memory clinic: study design and baseline findings of the MEMENTO cohort
Source: Alzheimers Res Ther. 2017 Aug 29;9:67. doi: 10.1186/s13195-017-0288-0 (PMC5576287; doi:10.1186/s13195-017-0288-0)
Supplement: Supplementary file 2 — Statistical significance of two-by-two cognitive categories comparisons of baseline characteristics distributions: the MEMENTO cohort. Table S2. Baseline characteristics by age group and sex: the MEMENTO cohort. Table S3. Association between baseline characteristics and number of copies of ε4 allele of APOE genotype: the MEMENTO cohort. (DOCX 47 kb) [file 13195_2017_288_MOESM2_ESM.docx]

Table S1. Statistical significance of 2 by 2 cognitive categories comparisons^[[1]](#footnote-1)^ of baseline characteristics distributions. The Memento cohort

|  | **1 : Isolated subjective Cognitive complaint (SCC) 2: Single domain aMCI 3. Multi-domain aMCI  4: Single domain naMCI 5.Multi-domain aMCI** | | | | | | | | | |
| --- | --- | --- | --- | --- | --- | --- | --- | --- | --- | --- |
|  | **1 vs. 2** | **1 vs. 3** | **1 vs. 4** | **1 vs. 5** | **2 vs. 3** | **2 vs. 4** | **2 vs. 5** | **3 vs. 4** | **3 vs. 5** | **4 vs. 5** |
| Women, % | 0.007 | 0.05 | NS | NS | NS | <0.001 | <0.001 | <0.001 | <0.001 | NS |
| Age in years, mean | 0.007 | <0.001 | 0.06 | 0.0005 | 0.094 | NS | NS | <0.001 | 0.087 | 0.096 |
| Baccalaureate and above,% | 0.09 | 0.05 | NS | 0.09 | NS | NS | NS | 0.03 | NS | NS |
| Diabetes,% | NS | NS | NS | NS | NS | NS | NS | NS | NS | NS |
| Hypertension,% | NS | NS | NS | NS | NS | NS | NS | NS | NS | NS |
| Dyslipidemia,% | NS | NS | NS | NS | NS | NS | NS | NS | NS | NS |
| History of cardiovascular disease,% | NS | NS | NS | NS | NS | NS | NS | NS | NS | NS |
| Apathy,% | 0.03 | 0.01 | NS | NS | NS | NS | NS | NS | NS | NS |
| Depression,% | NS | 0.05 | NS | NS | NS | NS | NS | NS | NS | 0.05 |
| Anxiety,% |  |  |  |  |  |  |  |  |  |  |
| Number of limitations at IADL,%  1  2 or more | NS | NS | NS | NS | NS | NS | NS | NS | NS | NS |
| SPPB score, mean | 0.0122 | <0.0001 | NS | 0.0045 | NS | NS | NS | 0.0041 | NS | NS |
| At least one epsilon 4 allele for APOE^[[2]](#footnote-2)^ carried, % | NS | NS | NS | NS | NS | NS | NS | NS | NS | NS |
| CDR sum of the box, mean | 0.0121 | <0.001 | NS | <0.001 | <0.001 | 0.0547 | NS | <0.001 | <0.001 | 0.0016 |
| MMSE score, mean | 0.016 | <0.001 | NS | <0.001 | <0.001 | NS | 0.0054 | <0.001 | <0.001 | <0.001 |
| Verbal fluency – Letter P, mean | 0.016 | <0.001 | <0.001 | <0.001 | <0.001 | NS | <0.001 | <0.001 | NS | <0.001 |
| Verbal fluency – Animals, mean | 0.0026 | <0.001 | <0.001 | <0.001 | <0.001 | NS | <0.001 | <0.001 | <0.001 | <0.001 |
| DMS48 – Immediate recall, mean | <0.001 | <0.001 | NS | NS | <0.001 | <0.001 | <0.001 | <0.001 | <0.001 | NS |
| Praxis total score, mean | NS | <0.001 | <0.001 | <0.001 | <0.001 | 0.0004 | <0.001 | <0.001 | 0.0171 | <0.001 |
| TMT A time in sec, mean | NS | <0.001 | 0.0005 | <0.001 | <0.001 | 0.0097 | <0.001 | <0.001 | NS | <0.001 |
| TMT B time in sec, mean | NS | <0.001 | 0.0561 | <0.001 | <0.001 | NS | <0.001 | <0.001 | 0.0073 | <0.001 |
| FCSRT total immediate free recalls, mean | <0.001 | <0.001 | NS | NS | <0.001 | <0.001 | 0.0003 | <0.001 | <0.001 | <0.001 |
| FCSRT total free & cued delayed recalls,mean | <0.001 | <0.001 | NS | NS | <0.001 | <0.001 | <0.001 | <0.001 | <0.001 | NS |

Table S1 (continued). Statistical significance of 2 by 2 cognitive categories comparisons^[[3]](#footnote-3)^ of baseline characteristics distributions. The Memento cohort

|  | **1 : Isolated subjective Cognitive complaint (SCC) 2: Single domain aMCI 3. Multi-domain aMCI  4: Single domain naMCI 5.Multi-domain aMCI** | | | | | | | | | |
| --- | --- | --- | --- | --- | --- | --- | --- | --- | --- | --- |
|  | **1 vs. 2** | **1 vs. 3** | **1 vs. 4** | **1 vs. 5** | **2 vs. 3** | **2 vs. 4** | **2 vs. 5** | **3 vs. 4** | **3 vs. 5** | **4 vs. 5** |
| Digit span standardized score, mean | NS | <0.001 | 0.0005 | <0.001 | <0.001 | 0.0395 | <0.001 | <0.001 | 0.0074 | <0.001 |
| Rey figure immediate copy score, mean | NS | <0.001 | NS | <0.001 | <0.001 | NS | 0.0089 | <0.001 | NS | <0.001 |
| Rey figure 3 minutes copy score, mean | <0.001 | <0.001 | NS | <0.001 | <0.001 | <0.001 | <0.001 | <0.001 | <0.001 | <0.001 |
| FAB^[[4]](#footnote-4)^ score, mean | NS | <0.001 | <0.001 | <0.001 | <0.001 | <0.001 | <0.001 | <0.001 | NS | <0.001 |
| DO80 score, mean | NS | <0.001 | NS | 0.003 | <0.001 | NS | NS | <0.001 | 0.001 | 0.0334 |
| Hippocampal volume right in cm^3^, mean | 0.0355 | <0.001 | NS | 0.0269 | 0.0022 | 0.0722 | NS | <0.001 | <0.001 | 0.0602 |
| Hippocampal volume left in cm3, mean | 0.0021 | <0.001 | NS | 0.0025 | NS | 0.0109 | NS | <0.001 | 0.0097 | 0.0152 |
| Brain parenchymal fraction in %, mean | NS | <0.001 | NS | 0.0026 | 0.0134 | NS | NS | <0.001 | NS | 0.0015 |
| White matter lesions volume in cm3, mean | 0.0571 | <0.001 | NS | NS | 0.1516 | NS | NS | 0.0021 | 0.0106 | NS |
| Cortical thickness right in mm, mean | NS | <0.001 | NS | 0.0057 | 0.0201 | NS | NS | <0.001 | 0.0973 | 0.0610 |
| Cortical thickness left in mm, mean | NS | <0.001 | NS | 0.0039 | 0.0485 | NS | NS | 0.0004 | NS | 0.0138 |
| Angular & parietalinf right FDG uptake, mean | NS | <0.001 | NS | 0.0059 | 0.0002 | NS | NS | <0.001 | 0.0132 | 0.0071 |
| Parietalinf_left FDG uptake, mean | NS | <0.001 | NS | 0.0342 | <0.001 | NS | NS | <0.001 | 0.0063 | 0.0097 |
| Precuneus&CingulumPost left FDG uptake, mean | NS | <0.001 | NS | 0.0040 | 0.0002 | NS | NS | <0.001 | 0.0093 | 0.0054 |
| Temporal_Inf Left FDG uptake, mean | NS | <0.001 | NS | 0.0152 | <0.001 | NS | 0.0276 | <0.001 | 0.0367 | 0.0084 |
| Temporal_Inf Right FDG uptake, mean | NS | <0.001 | NS | 0.0188 | 0.0022 | NS | NS | <0.001 | 0.0023 | NS |

Table S2. Baseline characteristics by age group and sex. The Memento cohort

|  | AGE (IN YEARS) | | | | |  | Sex | |  |
| --- | --- | --- | --- | --- | --- | --- | --- | --- | --- |
|  | <60 | [60-69] | [70-79] | ≥80 | P value^^[[5]](#footnote-5)^^ | | Men | Women | P value^^[[6]](#footnote-6)^^ |
| N | 245 | 739 | 1014 | 325 |  | | 885 | 1438 |  |
| Women, % | 65.4 | 62.4 | 61.5 | 58.8 | 0.43 | | - | - |  |
| Age in years, mean (SD) | 53.0 (5.1) | 65.6 (2.7) | 74.9 (2.8) | 83.0 (2.6) | <0.0001 | | 71.3 (8.4) | 70.6 (8.9) | 0.08 |
| Baccalaureate and above,% | 52.1 | 56.1 | 56.7 | 48.3 | 0.04 | | 59.8 | 51.8 | 0.0002 |
| Diabetes,% | 7.8 | 8.0 | 7.5 | 5.5 | 0.56 | | 10.0 | 5.8 | 0.0002 |
| Hypertension,% | 17.3 | 29.0 | 37.4 | 45.5 | <10^-4^ | | 38.5 | 30.4 | 0.0002 |
| Dyslipidemia,% | 19.3 | 23.0 | 32.9 | 29.5 | <10^-4^ | | 30.9 | 25.9 | 0.01 |
| History of cardiovascular disease,% | 5.4 | 8.3 | 12.9 | 21.4 | <10^-4^ | | 18.0 | 8.0 | <10^-4^ |
| Apathy,% | 26.5 | 17.4 | 14.2 | 19.9 | 0.0002 | | 20.0 | 15.5 | 0.01 |
| Depression,% | 54.2 | 37.6 | 27.0 | 31.4 | <10^-4^ | | 30.8 | 35.3 | 0.03 |
| Anxiety,% | 56.4 | 49.2 | 34 .4 | 38.9 | <10^-4^ | | 35.5 | 45.6 | <10^-4^ |
| Number of limitations at IADL^^[[7]](#footnote-7)^^,%  1  2 or more | 6.5 1.3 | 6.9 0.9 | 12.3 2.4 | 14.2 7.8 | <10^-4^ | | 6.8 3.8 | 12.4 1.8 | <10^-4^ |
| SPPB score, mean (SD) | 11.3 (1.0) | 10.9 (1.7) | 10.5 (1.8) | 9.2 (2.3) | <10^-4^ | | 10.7 (1.7) | 10.4 (2.0) | 0.0005 |
| At least one epsilon 4 allele for APOE carried, % | 31.0 | 31.6 | 29.4 | 27.4 | 0.15 | | 31.9 | 28.8 | 0.29 |
| CDR sum of the box, mean (SD) | 0.56 (0.52) | 0.54 (0.59) | 0.57 (0.71) | 0.87 (0.94) | <0.0001 | | 0.65 (0.74) | 0.57 (0.69) | 0.0028 |
| MMSE score, mean (SD) | 28.2 (1.8) | 28.2 (1.7) | 27.8 (2.0) | 27.2 (2.) | <0.0001 | | 27.9 (1.9) | 27.9 (2.0) | 0.45 |
| Verbal fluency – Letter P, mean (SD) | 20.5 (7.5) | 21.3(7.4) | 20.1 (7.0) | 19.1 (7.0) | <0.0001 | | 19.9 (7.3) | 20.7 (7.2) | 0.02 |
| Verbal fluency – Animals, mean (SD) | 29.6 (9.1) | 30.0 (8.7) | 28.0 (8.9) | 24.3 (7.6) | <0.0001 | | 28.3 (9.0) | 28.3 (8.8) | 0.91 |
| DMS48 – Immediate recall, mean (SD) | 45.1 (3.9) | 45.4 (3.1) | 44.7 (4.2) | 42.9 (4.9) | <0.0001 | | 44.4 (4.2) | 44.8 (4.0) | 0.03 |
| Praxis total score, mean (SD) | 22.3 (1.3) | 22.2 (1.3) | 21.7 (1.6) | 21.1 (2.0) | <0.0001 | | 22.0 (1.5) | 21.8 (1.6) | 0.004 |
| TMT A time in sec, mean(SD) | 1.6 (0.67) | 1.8 (0.67) | 2.18 (0.86) | 2.63 (1.41) | <0.0001 | | 2.0 (0.87) | 2.1 (0.98) | 0.15 |
| TMT B time in sec, mean (SD) | 3.9 (2.2) | 4.3 (4.1) | 5.6 (4.4) | 6.8 (5.7) | <0.0001 | | 5.0 (3.7) | 5.3 (4.8) | 0.19 |
| FCSRT total immediate free recalls, mean (SD) | 28.4 (7.2) | 27.9 (7.5) | 25.5 (8.2) | 20.7 (9.2) | <0.0001 | | 24.2 (7.8) | 27.0 (8.5) | <10^-4^ |
| FCSRT total free & cued delayed recalls, mean (SD) | 15.0 (2.1) | 15.2 (1.9) | 14.9 (2.2) | 14.0 (3.0) | <0.0001 | | 14.6 (2.3) | 15.1 (2.2) | <10^-4^ |
| Digit span standardized score, mean (SD) | 8.7 (3.0) | 9.7 (2.9) | 10.4 (3.2) | 9.9 (3.2) | <0.0001 | | 10.0 (3.2) | 9.8 (3.1) | 0.11 |

Table S2 (continued). Baseline characteristics by age group and sex. The Memento cohort

|  | AGE (IN YEARS) | | | |  | GENDER | |  |
| --- | --- | --- | --- | --- | --- | --- | --- | --- |
|  | <=60 | ]60-69] | ]70-79] | ≥80 | Pvalue ^^[[8]](#footnote-8)^^ | Male | Female | P value^^[[9]](#footnote-9)^^ |
| N | 245 | 739 | 1014 | 325 |  | 885 | 1438 |  |
| Rey figure immediate copy score, mean (SD^^[[10]](#footnote-10)^^) | 33.8 (2.9) | 33.6 (3.4) | 32.6 (4.6) | 31.5 (5.6) | <0.0001 | 33.2 (3.9) | 32.7 (4.6) | 0.008 |
| Rey figure 3 minutes copy score, mean (SD) | 17.0 (6.6) | 16.6 (6.5) | 14.7 (7.1) | 11.3 (6.5) | <0.0001 | 16.2 (7.2) | 14.4 (6.8) | <0.0001 |
| FAB score, mean (SD) | 16.6 (1.6) | 16.6 (1.6) | 15.9 (1.9) | 15.4 (2.2) | <0.0001 | 16.0 (1.9) | 16.2 (1.9) | 0.01 |
| DO80 score, mean (SD) | 78.3 (5.4) | 79.0 (1.6) | 78.6 (3.4) | 77.9 (3.3) | <0.0001 | 78.5 (4.3) | 78.7 (2.4) | 0.14 |
| Hippocampal volume right in cm^3^, mean (SD) | 3.00 (0.30) | 2.88 (0.37) | 2.71 (0.40) | 2.41 (0.44) | <0.0001 | 2.84 (0.43) | 2.71 (0.41) | <0.0001 |
| Hippocampal volume left in cm3, mean (SD) | 2.88 (0.34) | 2.80 (0.37) | 2.61 (0.38) | 2.32 (0.43) | <0.0001 | 2.75 (0.41) | 2.60 (0.40) | <0.0001 |
| Brain parenchymal fraction in %, mean (SD) | 82.8 (1.2) | 82.0 (1.2) | 81.1 (1.1) | 80.5 (1.0) | <0.0001 | 81.1 (1.2) | 81.7 (1.4) | <0.0001 |
| White matter lesions volume in cm3, mean (SD) | 3.2 (5.5) | 6.9 (11.1) | 12.0 (14.3) | 16.1 (16.9) | <0.0001 | 11.3 (14.6) | 9.7 (13.2) | 0.01 |
| Cortical thickness right in mm, mean (SD) | 2.38 (0.10) | 2.35 (0.10) | 2.31 (0.10) | 2.27 (0.11) | <0.0001 | 2.31 (0.11) | 2.34 (0.11) | <0.0001 |
| Cortical thickness left in mm, mean (SD) | 2.39 (0.10) | 2.35 (0.10) | 2.31 (0.10) | 2.27 (0.11) | <0.0001 | 2.31 (0.12) | 2.34 (0.10) | <0.0001 |
| Angular & parietalinf right FDG uptake, mean (SD) | 1.86 (0.19) | 1.79 (0.22) | 1.73 (0.20) | 1.61 (0.19) | <0.0001 | 1.67 (0.20) | 1.79 (0.21) | <0.0001 |
| Parietalinf_left FDG uptake, mean (SD) | 1.85 (0.19) | 1.77 (0.21) | 1.68 (0.19) | 1.57 (0.16) | <0.0001 | 1.65 (0.20) | 1.76 (0.20) | <0.0001 |
| Precuneus&CingulumPost left FDG uptake, mean(SD) | 2.07 (0.22) | 1.99 (0.24) | 1.92 (0.22) | 1.80 (0.22) | <0.0001 | 1.88 (0.23) | 1.99 (0.23) | <0.0001 |
| Temporal_Inf Left FDG uptake, mean (SD) | 1.72 (0.16) | 1.66 (0.17) | 1.59 (0.16) | 1.51 (0.15) | <0.0001 | 1.57 (0.17) | 1.65 (0.17) | <0.0001 |
| Temporal_Inf Right FDG uptake, mean (SD) | 1.72 (0.16) | 1.66 (0.18) | 1.62 (0.17) | 1.55 (0.15) | <0.0001 | 1.59 (0.16) | 1.67 (0.17) | <0.0001 |

Table S3. Association between baseline characteristics and number of copies of epsilon 4 allele of APOE genotype. The Memento cohort

|  | **NUMBER OF EPISLON 4 ALLELES FOR APOE^[[11]](#footnote-11)^ GENOTYPE** | | |  |
| --- | --- | --- | --- | --- |
|  | 0 | 1 | 2 | P^[[12]](#footnote-12)^ |
| N | 1532 | 581 | 75 |  |
| Women, % | 62.8 | 59.6 | 57.3 | 0.28 |
| Age in years, mean (SD) | 71.0 (8.8) | 70.7 (8.5) | 68.5 (7.9) | <0.0001 |
| Baccalaureate and above,% | 55.1 | 53.1 | 57.3 | 0.63 |
| Diabetes,% | 8.4 | 5.7 | 5.3 | 0.08 |
| Hypertension,% | 34.0 | 32.5 | 40.0 | 0.40 |
| Dyslipidemia,% | 25.1 | 34.8 | 42.7 | <0.001 |
| History of cardiovascular disease,% | 11.7 | 11.6 | 14.9 | 0.71 |
| Apathy,% | 16.5 | 17.7 | 28.2 | 0.04 |
| Depression,% | 32.4 | 35.7 | 38.0 | 0.29 |
| Anxiety,% | 40.4 | 43.7 | 55.6 | 0.03 |
| Number of limitations at IADL,%  1  2 or more | 11.2 2.6 | 8.1 2.5 | 8.7 4.3 | 0.26 |
| SPPB score, mean (SD) | 10.5 (0.05) | 10.6 (0.07) | 10.2 (0.21) | 0.64 |
| CDR sum of the box, mean (SE) | 0.57 (0.02) | 0.68 (0.03) | 0.87 (0.08) | <0.0001 |
| MMSE score, mean (SE) | 28.0 (0.05) | 27.6 (0.08) | 27.0 (0.21) | <0.0001 |
| Verbal fluency – Letter P, mean (SE) | 20.3 (0.17) | 20.5 (0.28) | 20.9 (0.78) | 0.61 |
| Verbal fluency – Animals, mean (SE) | 28.6 (0.22) | 27.7 (0.36) | 28.0 (0.98) | 0.10 |
| DMS48 – Immediate recall, mean (SE) | 44.9 (0.10) | 44.3 (0.17) | 43.6 (0.46) | 0.004 |
| Praxis total score, mean (SE) | 21.9 (0.04) | 21.8 (0.06) | 21.8 (0.18) | 0.34 |
| TMT A time in sec, mean(SE) | 2.03 (0.03) | 2.1 (0.04) | 2.1 (0.11) | 0.03 |
| TMT B time in sec, mean (SE) | 4.9 (0.11) | 5.7 (0.18) | 6.4 (0.50) | <0.0001 |
| FCSRT total immediate free recalls, mean (SE) | 26.8 (0.19) | 24.7 (0.32) | 20.2 (0.89) | <0.0001 |
| FCSRT total free & cued delayed recalls, mean(SE) | 15.1 (0.06) | 14.6 (0.09) | 13.1 (0.25) | <0.0001 |
| Digit span standardized score, mean (SE) | 10.0 (0.08) | 9.7 (0.13) | 9.9 (0.35) | 0.25 |

Table S3 (continued). Association between baseline characteristics and number of copies of epsilon 4 allele of APOE genotype. The Memento cohort

|  | **NUMBER OF EPSILON 4 ALLELES FOR APOE^[[13]](#footnote-13)^ GENOTYPE** | | |  |
| --- | --- | --- | --- | --- |
|  | 0 | 1 | 2 | p^[[14]](#footnote-14)^ |
| N | 1532 | 581 | 75 |  |
| Rey figure immediate copy score, mean (SE) | 33.0 (0.11) | 32.7 (0.17) | 32.1 (0.48) | 0.15 |
| Rey figure 3 minutes copy score, mean (SE) | 15.6 (0.18) | 14.5 (0.29) | 12.4 (0.79) | <0.0001 |
| FAB score, mean (SE) | 16.2 (0.05) | 16.0 (0.08) | 15.8 (0.21) | <0.0001 |
| DO80 score, mean (SE) | 78.6 (0.08) | 78.5 (0.14) | 78.9 (0.38) | 0.45 |
| Hippocampal volume right in cm^3^, mean (SE) | 2.77 (0.01) | 2.75 (0.02) | 2.60 (0.05) | 0.0007 |
| Hippocampal volume left in cm3, mean (SE) | 2.68 (0.01) | 2.64 (0.02) | 2.54 (0.04) | 0.0019 |
| Brain parenchymal fraction in %, mean (SE) | 81.5 (0.03) | 81.5 (0.03) | 81.6 (0.13) | 0.70 |
| White matter lesions volume in cm3, mean (SE) | 10.0 (0.37) | 9.9 (0.60) | 17.8 (1.66) | <0.0001 |
| Cortical thickness right in mm, mean (SE) | 2.33 (0.003) | 2.32 (0.004) | 2.30 (0.01) | 0.09 |
| Cortical thickness left in mm, mean (SE) | 2.33 (0.003) | 2.32 (0.004) | 2.31 (0.01) | 0.25 |
| Angular & parietalinf right FDG uptake, mean (SE) | 1.76 (0.006) | 1.72 (0.01) | 1.59 (0.03) | <0.0001 |
| Parietalinf_left FDG uptake, mean (SE) | 1.73 (0.006) | 1.69 (0.01) | 1.59 (0.03) | <0.0001 |
| Precuneus&CingulumPost left FDG uptake, mean(SE) | 1.96 (0.007) | 1.93 (0.01) | 1.78 (0.03) | <0.0001 |
| Temporal_Inf Left FDG uptake, mean (SE) | 1.62 (0.01) | 1.60 (0.01) | 1.54 (0.02) | 0.0006 |
| Temporal_Inf Right FDG uptake, mean (SE) | 1.65 (0.01) | 1.62 (0.01) | 1.52 (0.02) | <0.0001 |

1. P value adjusted for center, sex, education level and clinical dementia rating scale score (0 vs. 0.5) computed from polytomous logistic regression model for categorical variables, generalized linear model for continuous variables [↑](#footnote-ref-1)
2. APOE=Apolipoprotein E; CDR=Clinical dementia rating; DMS= Delayed Matching to Sample, FCSRT=Free and Cued Selective Reminding Test, IADL = Instrumental Activities of Daily Living; SD=Standard Deviation; SPPB=Short Physical Performance Battery; TMT=Trail Making Test [↑](#footnote-ref-2)
3. P value adjusted for center, sex, education level and clinical dementia rating scale score (0 vs. 0.5) computed from polytomous logistic regression model for categorical variables, generalized linear model for continuous variables [↑](#footnote-ref-3)
4. DO="Dénomination d'Objet" (Object naming); FAB= Frontal Assessment Battery, FDG=FluoroDésoxyGlucose; Inf=Inferior, [↑](#footnote-ref-4)
5. P value adjusted for center, sex, education level and clinical dementia rating scale score (0 vs. 0.5) computed from polytomous logistic regression model for categorical variables, generalized linear model for continuous variables [↑](#footnote-ref-5)
6. P value adjusted for center, age, education level and clinical dementia rating scale score (0 vs. 0.5) computed from polytomous logistic regression model for categorical variables, generalized linear model for continuous variables [↑](#footnote-ref-6)
7. APOE=Apolipoprotein E; CDR=Clinical dementia rating; DMS= Delayed Matching to Sample, FCSRT=Free and Cued Selective Reminding Test, IADL = Instrumental Activities of Daily Living; SD=Standard Deviation; SPPB=Short Physical Performance Battery; TMT=Trail Making Test [↑](#footnote-ref-7)
8. P value adjusted for center, gender, education level and clinical dementia rating scale score (0 vs. 0.5) computed from polytomous logistic regression model for categorical variables, generalized linear model for continuous variables [↑](#footnote-ref-8)
9. P value adjusted for center, age, education level and clinical dementia rating scale score (0 vs. 0.5) computed from polytomous logistic regression model for categorical variables, generalized linear model for continuous variables [↑](#footnote-ref-9)
10. DO="Dénomination d'Objet" (Object naming); FAB= Frontal Assessment Battery, FDG=FluoroDésoxyGlucose; Inf=Inferior, SD=Standard Deviation [↑](#footnote-ref-10)
11. APOE=Apolipoprotein E; CDR=Clinical dementia rating; DMS= Delayed Matching to Sample; IADL = Instrumental Activities of Daily Living; SD=Standard Deviation; SE=Standard Error; SPPB=Short Physical Performance Battery; TMT=Trail Making Test [↑](#footnote-ref-11)
12. P value adjusted for center, age, gender, education level and clinical dementia rating scale score (0 vs. 0.5) computed from polytomous logistic regression model for categorical variables, generalized linear model for continuous variables [↑](#footnote-ref-12)
13. APOE=Apolipoprotein E; DO="Dénomination d'Objet" (Object naming); FAB= Frontal assessment Battery; FDG=FluoroDésoxyGlucose; SE=Standard Error [↑](#footnote-ref-13)
14. P value adjusted for center, age, gender, education level and clinical dementia rating scale score (0 vs. 0.5) computed from polytomous logistic regression model for categorical variables, generalized linear model for continuous variables [↑](#footnote-ref-14)
