# Supplementary material for: Cognitive and imaging markers in non-demented subjects attending a memory clinic: study design and baseline findings of the MEMENTO cohort
Source: Alzheimers Res Ther. 2017 Aug 29;9:67. doi: 10.1186/s13195-017-0288-0 (PMC5576287; doi:10.1186/s13195-017-0288-0)
Supplement: Supplementary file 3 — Memento Study Group list. (DOCX 28 kb) [file 13195_2017_288_MOESM3_ESM.docx]

Bertrand, Accart, Memory Resource and Research Centre of Lille, CHRU de Lille, Lille, France

Geneviève, Achdjibachdian, Memory Resource and Research Centre of Marseille, CHU de Marseille, Marseille, France

Isabelle, Addra, CIC-1401 Clinical Epidemiology, CHU Bordeaux, Bordeaux, France

Sarah, Adjal, Memory Resource and Research Centre of Bordeaux, CHU Bordeaux, Bordeaux, France

Timothée, Albasser, Memory Resource and Research Centre of Strasbourg, CHRU de Strasbourg, Strasbourg, France

Michèle, Allard, Memory Resource and Research Centre of Bordeaux, CHU de Bordeaux, Bordeaux France

Sandrine, Andrieu, Memory Resource and Research Centre of Toulouse, CHU de Toulouse, Toulouse, France

Cédric, Annweiller, Memory Resource and Research Centre of Angers, CHU d’Angers, Angers, France

Pierre, Anthony, Memory Resource and Research Centre of Colmar, Colmar, France

Elise, Antoine, Memory Resource and Research Centre of Lyon, Hospices Civils de Lyon, Lyon, France

Jean-Paul, Armspach, Memory Resource and Research Centre of Strasbourg, CHRU de Strasbourg, Strasbourg, France

Christine, Astier, Memory Resource and Research Centre of Strasbourg, CHRU de Strasbourg, Strasbourg, France

Vanessa, Auberti, Memory Resource and Research Centre of Bordeaux, CHU de Bordeaux, Bordeaux, France

Christelle, Audrain, Institute of Memory and Alzheimer's Disease (IM2A), France and Brain and Spine Institute (ICM), France UMR S 1127, Department of Neurology, AP-HP, Pitié-Salpêtrière University Hospital, Sorbonne Universities, Pierre et Marie Curie University, Paris, France

Alexandre, Augier, Memory Clinic of Avicenne, Hôpital Avicenne, Bobigny, France

Sophie, Auriacombe, Memory Resource and Research Centre of Bordeaux, CHU de Bordeaux, Bordeaux, France

John, Avet, Memory Resource and Research Centre of Saint-Etienne, CHU de Saint-Etienne, Saint-Etienne, France

Romain, Bachelet, Memory Resource and Research Centre of Lyon, Hospices Civils de Lyon, Lyon, France

Olivier, Bailon, Memory Clinic of Avicenne, Hôpital Avicenne, Bobigny, France

Hélène, Bansard, Memory Resource and Research Centre of Tours, CHRU de Tours, Tours, France

Laurent, Baranti, Memory Resource and Research Centre of Tours, CHRU de Tours, Tours, France

Fabrice-Guy, Barral, Memory Resource and Research Centre of Saint-Etienne, CHU de Saint-Etienne, Saint-Etienne, France

Jean, Barré, Memory Resource and Research Centre of Angers, CHU d’Angers, Angers, France

Yoan, Barsznica, Memory Resource and Research Centre of Besançon, Besançon, France

Annick, Barthelaix, Memory Resource and Research Centre of Angers, CHU d’Angers, Angers, France

Laurie, Barthelemi, Memory Resource and Research Centre of Montpellier, CHU de Montpellier, Montpellier, France

Fanny, Barthelemy, Memory Resource and Research Centre of Marseille, CHU de Marseille, Marseille, France

Anthony, Bathsavanis, Memory Resource and Research Centre of Lyon, Hospices Civils de Lyon, Lyon, France

Vanessa, Baudiffier, Memory Resource and Research Centre of Poitiers, CHU de Poitiers, Poitiers, France

Sophie, Bayer, Memory Resource and Research Centre of Strasbourg, CHRU de Strasbourg, Strasbourg, France

Catherine, Bayle, Memory Resource and Research Centre of Paris Broca, AP-HP, Paris, France

Mélinda, Beaudenon, Memory Resource and Research Centre of Angers, CHU d’Angers, Angers, France

Emilie, Beaufils, Memory Resource and Research Centre of Tours, CHRU de Tours, Tours, France

Yannick, Bejot, Memory Resource and Research Centre of Dijon, CHU Dijon Bourgogne, Dijon, France

Sandrine, Belkadi, Memory Resource and Research Centre of Poitiers, CHU de Poitiers, Poitiers, France

Julie, Bellet, Memory Resource and Research Centre of Lille, CHRU de Lille, Lille, France

Marwa, Ben, Yacoub, Memory Clinic of Avicenne, Hôpital Avicenne, Bobigny, France

Habib, Benali, Institute of Memory and Alzheimer's Disease (IM2A), France and Brain and Spine Institute (ICM), France UMR S 1127, Department of Neurology, AP-HP, Pitié-Salpêtrière University Hospital, Sorbonne Universities, Pierre et Marie Curie University, Paris, France

Karim, Bennys, Memory Resource and Research Centre of Montpellier, CHU de Montpellier, Montpellier, France

Nadine, Bensoussan, Memory Resource and Research Centre of Marseille, CHU de Marseille, Marseille, France

Géraldine, Bera, Laboratoire d'Imagerie Biomédicale, Sorbonne Universités, UPMC Univ Paris 06, Inserm U 1146, CNRS UMR 7371, F-75006 Paris, France NeuroSpin, I2BM, Commissariat à l'Energie Atomique, France

Eric, Berger, Memory Resource and Research Centre of Besançon, Besançon, France

Juliette, Berger, Memory Resource and Research Centre of Clermont-Ferrand, CHU de Clermont-Ferrand, Clermont-Ferrand, France

Marc, G, Berger, Memory Resource and Research Centre of Clermont-Ferrand, CHU de Clermont-Ferrand, Clermont-Ferrand, France

Georgette, Berlier, Memory Resource and Research Centre of Saint-Etienne, CHU de Saint-Etienne, Saint-Etienne, France

Laëtitia, Berly, Memory Resource and Research Centre of Strasbourg, CHRU de Strasbourg, Strasbourg, France

Hassan, Berrissoul, Memory Resource and Research of Amiens, CHU Amiens Picardie, Amiens, France

Marie-Camille, Berthel, Memory Resource and Research Centre of Colmar, Colmar, France

Véronique, Berthier, Memory Resource and Research Centre of Lyon, Hospices Civils de Lyon, Lyon, France

François, Bertin-Hugault, Memory Resource and Research Centre of Lyon, Hospices Civils de Lyon, Lyon, France

François-Xavier, Bertrand, Memory Resource and Research Centre of Nantes, CHU de Nantes, Nantes, France

Guillaume, Bertrand, Memory Clinic of Avicenne, Hôpital Avicenne, Bobigny, France

Anaïck, Besozzi, Memory Resource and Research Centre of Nancy, CHU de Nancy, Nancy, France

Christelle, Betogliati-Filleau, Memory Resource and Research Centre of Nice, CHU de Nice, Nice, France

Catherine, Beze, Memory Resource and Research Centre of Tours, CHRU de Tours, Tours, France

Mathias, Bilger, Memory Resource and Research Centre of Strasbourg, CHRU de Strasbourg, Strasbourg, France

Sandrine, Bioux, Memory Resource and Research Centre of Rouen, CHU de Rouen, Rouen, France

Elisa, Bittard, Memory Resource and Research Centre of Bordeaux, CHU de Bordeaux, Bordeaux, France

Ludovic, Blanchard, Memory Resource and Research Centre of Poitiers, CHU de Poitiers, Poitiers, France

Odile, Blanchet, Memory Resource and Research Centre of Angers, CHU d’Angers, Angers, France

Maryline, Blanchon, Memory Resource and Research Centre of Marseille, CHU de Marseille, Marseille, France

Evangéline, Bliaux, Memory Resource and Research Centre of Rouen, CHU de Rouen, Rouen, France

Pierre, Bohn, Memory Resource and Research Centre of Rouen, CHU de Rouen, Rouen, France

Stéphanie, Bombois, Memory Resource and Research Centre of Lille, CHRU de Lille, Lille, France

Alain, Bonafe, Memory Resource and Research Centre of Montpellier, CHU de Montpellier, Montpellier, France

Marie, Bonnet, Memory Resource and Research Centre of Bordeaux, CHU de Bordeaux, Bordeaux, France

Hélène, Bonnot, Memory Resource and Research Centre of Strasbourg, CHRU de Strasbourg, Strasbourg, France

Martine, Bordessoules, Memory Resource and Research Centre of Bordeaux, CHU de Bordeaux, Bordeaux, France

Nathalie, Bortone, Memory Resource and Research Centre of Nice, CHU de Nice, Nice, France

Amandine, Bossant, Memory Resource and Research Centre of Grenoble, CHU de Grenoble Alpes, Grenoble, France

Elodie, Bouaziz, Phar, Memory Resource and Research Centre of Paris Nord, AP-HP, Paris, France

Yasmina, Boudali, Memory Resource and Research Centre of Paris Broca, AP-HP, Paris, France

Laurie, Boukadida, Institute of Memory and Alzheimer's Disease (IM2A), France and Brain and Spine Institute (ICM), France UMR S 1127, Department of Neurology, AP-HP, Pitié-Salpêtrière University Hospital, Sorbonne Universities, Pierre et Marie Curie University, Paris, France

Justine, Boulanghien, Memory Resource and Research Centre of Montpellier, CHU de Montpellier, Montpellier, France

Clemence, Boully, Memory Resource and Research Centre of Paris Broca, AP-HP, Paris, France

Isabelle, Bourdel-Marchasson, Memory Resource and Research Centre of Bordeaux, CHU de Bordeaux, Bordeaux, France

Marie-France, Bourin, Memory Resource and Research Centre of Poitiers, CHU de Poitiers, Poitiers, France

Christophe, Bouvier, Coordinating Centre, CIC-1401 Clinical Epidemiology, Bordeaux, France

Serge, Bracard, Memory Resource and Research Centre of Nancy, CHU de Nancy, Nancy, France

Antoine, Brangier, Memory Resource and Research Centre of Angers, CHU d’Angers, Angers, France

Laëtitia, Breuilh, Memory Resource and Research Centre of Lille, CHRU de Lille, Lille, France

Lysiane, Brick, Memory Resource and Research Centre of Tours, CHRU de Tours, Tours, France

Marie-Laure, Brickert, Memory Resource and Research Centre of Colmar, Colmar, France

Pierre-Yves, Brillet, Memory Clinic of Avicenne, Hôpital Avicenne, Bobigny, France

Signe, Brinck, Memory Resource and Research Centre of Nice, CHU de Nice, Nice, France

Caroline, Buisson, Memory Resource and Research Centre of Bordeaux, CHU de Bordeaux, Bordeaux, France

Francine, Bury, Memory Resource and Research Centre of Colmar, Colmar, France

Laurence, Cadet, Memory Resource and Research Centre of Saint-Etienne, CHU de Saint-Etienne, Saint-Etienne, France

Julien, Cahors, Memory Resource and Research Centre of Nice, CHU de Nice, Nice, France

Laure, Caillard, Memory Resource and Research Centre of Paris Broca, AP-HP, Paris, France

Maria, Callejo, Plazas, Memory Resource and Research Centre of Nice, CHU de Nice, Nice, France

Fabienne, Calvas, Memory Resource and Research Centre of Toulouse, CHU de Toulouse, Toulouse, France

Sabine, Camara, Memory Resource and Research Centre of Colmar, Colmar, France

Aurore, Camoreyt, Memory Resource and Research Centre of Colmar, Colmar, France

Sandra, Campagne, Memory Resource and Research Centre of Marseille, CHU de Marseille, Marseille, France

Agnès, Camus, Memory Resource and Research Centre of Dijon, CHU Dijon Bourgogne, Dijon, France

Vincent, Camus, Memory Resource and Research Centre of Tours, CHRU de Tours, Tours, France

Sandrine, Canaple, Memory Resource and Research of Amiens, CHU Amiens Picardie, Amiens, France

Edith, Carneiro, Memory Resource and Research Centre of Toulouse, CHU de Toulouse, Toulouse, France

Sabine, Caron, Memory Resource and Research Centre of Lille, CHRU de Lille, Lille, France

Antoine, Carpentier, Memory Clinic of Avicenne, Hôpital Avicenne, Bobigny, France

Elise, Carré, Memory Resource and Research Centre of Lille, CHRU de Lille, Lille, France

Isabelle, Carrie, Memory Resource and Research Centre of Toulouse, CHU de Toulouse, Toulouse, France

Pascaline, Cassagnaud, Memory Resource and Research Centre of Lille, CHRU de Lille, Lille, France

Françoise, Cattin, Memory Resource and Research Centre of Besançon, Besançon, France

Valérie, Causse-Lemercier, Laboratoire d'Imagerie Biomédicale, Sorbonne Universités, UPMC Univ Paris 06, Inserm U 1146, CNRS UMR 7371, F-75006 Paris, France NeuroSpin, I2BM, Commissariat à l'Energie Atomique, France

Anne, Cavey, Memory Resource and Research Centre of Nice, CHU de Nice, Nice, France

Matthieu, Chabel, Memory Resource and Research Centre of Lille, CHRU de Lille, Lille, France

Ludivine, Chamard, Memory Resource and Research Centre of Besançon, Besançon, France

Stéphane, Chanalet, Memory Resource and Research Centre of Nice, CHU de Nice, Nice, France

Thierry, Chaptal, Memory Resource and Research Centre of Montpellier, CHU de Montpellier, Montpellier, France

Annik, Charnallet, Memory Resource and Research Centre of Grenoble, CHU de Grenoble Alpes, Grenoble, France

Hélène, Chartrel, Memory Resource and Research Centre of Angers, CHU d’Angers, Angers, France

Mathieu, Chastan, Memory Resource and Research Centre of Rouen, CHU de Rouen, Rouen, France

Rose-May, Chaudat, Memory Resource and Research Centre of Marseille, CHU de Marseille, Marseille, France

Sophie, Chauvelier, Memory Resource and Research Centre of Paris Broca, AP-HP, Paris, France

Valérie, Chauvire, Memory Resource and Research Centre of Angers, CHU d’Angers, Angers, France

Samia, Cheriet, Memory Resource and Research Centre of Toulouse, CHU de Toulouse, Toulouse, France

Sylvie, Chiron, Memory Resource and Research Centre of Colmar, Colmar, France

Gilles, Chopard, Memory Resource and Research Centre of Besançon, Besançon, France

Emilie, Chrétien, Memory Resource and Research Centre of Lyon, Hospices Civils de Lyon, Lyon, France

Dominique, Clamens, Memory Resource and Research Centre of Montpellier, CHU de Montpellier, Montpellier, France

Anthony, Clotagatide, Memory Resource and Research Centre of Saint-Etienne, CHU de Saint-Etienne, Saint-Etienne, France

Emmanuel, Cognat, Memory Resource and Research Centre of Paris Nord, AP-HP, Paris, France

Lora, Cohen, Memory Resource and Research Centre of Grenoble, CHU de Grenoble Alpes, Grenoble, France

Olivier, Colliot, Institute of Memory and Alzheimer's Disease (IM2A), France and Brain and Spine Institute (ICM), France UMR S 1127, Department of Neurology, AP-HP, Pitié-Salpêtrière University Hospital, Sorbonne Universities, Pierre et Marie Curie University, Paris, France

Jean-Marc, Constans, Memory Resource and Research of Amiens, CHU Amiens Picardie, Amiens, France

Elodie, Cordier, Memory Resource and Research Centre of Lille, CHRU de Lille, Lille, France

Marie-Hélène, Coste, Memory Resource and Research Centre of Lyon, Hospices Civils de Lyon, Lyon, France

Jean-Philippe, Cottier, Memory Resource and Research Centre of Tours, CHRU de Tours, Tours, France

François, Cotton, Memory Resource and Research Centre of Lyon, Hospices Civils de Lyon, Lyon, France

Pierre, Malick, Coulibaly, Memory Resource and Research Centre of Nice, CHU de Nice, Nice, France

Isabelle, Couret, Memory Resource and Research Centre of Montpellier, CHU de Montpellier, Montpellier, France

Françoise, Courtin, Memory Resource and Research of Amiens, CHU Amiens Picardie, Amiens, France

Olivier-François, Couturier, Memory Resource and Research Centre of Angers, CHU d’Angers, Angers, France

Pascale, Cowppli-Bony, Memory Resource and Research Centre of Bordeaux, CHU de Bordeaux, Bordeaux, France

Véronique, Cressot, Memory Resource and Research Centre of Bordeaux, CHU de Bordeaux, Bordeaux, France

Benjamin, Crétin, Memory Resource and Research Centre of Strasbourg, CHRU de Strasbourg, Strasbourg, France

Marie-Hélène, Criscione, Memory Clinic of Avicenne, Hôpital Avicenne, Bobigny, France

Laurie, Cuche, Memory Resource and Research Centre of Saint-Etienne, CHU de Saint-Etienne, Saint-Etienne, France

Audrey, Dalbo, Memory Resource and Research Centre of Bordeaux, CHU de Bordeaux, Bordeaux, France

Keren, Danaila, Memory Resource and Research Centre of Lyon, Hospices Civils de Lyon, Lyon, France

Sabine, Dantzer, Memory Resource and Research Centre of Strasbourg, CHRU de Strasbourg, Strasbourg, France

Frédérique, Darcourt, Memory Resource and Research Centre of Nice, CHU de Nice, Nice, France

Jacques, Darcourt, Memory Resource and Research Centre of Nice, CHU de Nice, Nice, France

Pascal, Dartois, Memory Resource and Research Centre of Lille, CHRU de Lille, Lille, France

Ana-Maria, Dascalita, Memory Resource and Research Centre of Saint-Etienne, CHU de Saint-Etienne, Saint-Etienne, France

Marie-Claude, Daudon, Memory Resource and Research Centre of Saint-Etienne, CHU de Saint-Etienne, Saint-Etienne, France

Francesca, De, Anna, Memory Resource and Research Centre of Marseille, CHU de Marseille, Marseille, France

Virginie, de, Beco, Memory Clinic of Avicenne, Hôpital Avicenne, Bobigny, France

Xavier, de, Petigny, Memory Resource and Research Centre of Strasbourg, CHRU de Strasbourg, Strasbourg, France

Delphine, De, Verbizier-Lonjon, Memory Resource and Research Centre of Montpellier, CHU de Montpellier, Montpellier, France

Marielle, Decousus, Memory Resource and Research Centre of Saint-Etienne, CHU de Saint-Etienne, Saint-Etienne, France

Isabelle, Defouilloy, Memory Resource and Research of Amiens, CHU Amiens Picardie, Amiens, France

Cécile, Delaunay-Bretaut, Memory Resource and Research Centre of Angers, CHU d’Angers, Angers, France

Xavier, Delbeuck, Memory Resource and Research Centre of Lille, CHRU de Lille, Lille, France

Melissa, Delhommeau, Memory Resource and Research Centre of Nice, CHU de Nice, Nice, France

Christine, Delmaire, Memory Resource and Research Centre of Lille, CHRU de Lille, Lille, France

Floriane, Delphin-Combe, Memory Resource and Research Centre of Lyon, Hospices Civils de Lyon, Lyon, France

Julien, Delrieu, Memory Resource and Research Centre of Toulouse, CHU de Toulouse, Toulouse, France

Catherine, Demuyinck, Memory Resource and Research Centre of Strasbourg, CHRU de Strasbourg, Strasbourg, France

Vincent, Deramecourt, Memory Resource and Research Centre of Lille, CHRU de Lille, Lille, France

Hervé, Deramond, Memory Resource and Research of Amiens, CHU Amiens Picardie, Amiens, France

Virginie, Derenaucourt, Memory Resource and Research Centre of Lille, CHRU de Lille, Lille, France

Thomas, Desmidt, Memory Resource and Research Centre of Tours, CHRU de Tours, Tours, France

Marie-Dominique, Desruet, Memory Resource and Research Centre of Grenoble, CHU de Grenoble Alpes, Grenoble, France

Julien, Detour, Memory Resource and Research Centre of Strasbourg, CHRU de Strasbourg, Strasbourg, France

Audrey, Deudon, Memory Resource and Research Centre of Nice, CHU de Nice, Nice, France

Viviane, Derreux, Coordinating Centre, CIC-1401 Clinical Epidemiology, Administrative Assistant, France

Agnès, Devendeville, Memory Resource and Research of Amiens, CHU Amiens Picardie, Amiens, France

Laure, Di, Bitonto, Memory Resource and Research Centre of Strasbourg, CHRU de Strasbourg, Strasbourg, France

Sally, Dia, Memory Resource and Research Centre of Colmar, Colmar, France

Mira, Didic, Memory Resource and Research Centre of Marseille, CHU de Marseille, Marseille, France

Maritchu, Doireau, Memory Resource and Research Centre of Bordeaux, CHU de Bordeaux, Bordeaux, France

Marie-Thérèse, Dorier, Memory Resource and Research Centre of Besançon, Besançon, France

Antonio, Dos, Santos, Institute of Memory and Alzheimer's Disease (IM2A), France and Brain and Spine Institute (ICM), France UMR S 1127, Department of Neurology, AP-HP, Pitié-Salpêtrière University Hospital, Sorbonne Universities, Pierre et Marie Curie University, Paris, France

Patrice, Douillet, Memory Resource and Research Centre of Montpellier, CHU de Montpellier, Montpellier, France

Déborah, Drai, Memory Resource and Research Centre of Lyon, Hospices Civils de Lyon, Lyon, France

Foucaud, Du, Boisgueheneuc, Memory Resource and Research Centre of Poitiers, CHU de Poitiers, Poitiers, France

Delphine, Dubail, Memory Resource and Research Centre of Paris Broca, AP-HP, Paris, France

Sandrine, Duchez, Memory Resource and Research Centre of Saint-Etienne, CHU de Saint-Etienne, Saint-Etienne, France

Nathalie, Dufay, Memory Resource and Research Centre of Lyon, Hospices Civils de Lyon, Lyon, France

Sophie, Dulhoste, Memory Resource and Research Centre of Bordeaux, CHU de Bordeaux, Bordeaux, France

Julien, Dumont, Memory Resource and Research Centre of Lille, CHRU de Lille, Lille, France

Julien, Dumurgier, Memory Resource and Research Centre of Paris Nord, AP-HP, Paris, France

Mélanie, Dupin, Memory Resource and Research Centre of Saint-Etienne, CHU de Saint-Etienne, Saint-Etienne, France

Diane, Dupuy, Memory Resource and Research of Amiens, CHU Amiens Picardie, Amiens, France

Emmanuelle, Durand, Memory Resource and Research Centre of Bordeaux, CHU de Bordeaux, Bordeaux, France

Emmanuelle, Duron, Memory Resource and Research Centre of Paris Broca, AP-HP, Paris, France

Inna, Dygai-Cochet, Memory Resource and Research Centre of Dijon, CHU Dijon Bourgogne, Dijon, France

Véronique, Eder, Memory Clinic of Avicenne, Hôpital Avicenne, Bobigny, France

Emmanuelle, Ehrhard, Memory Resource and Research Centre of Strasbourg, CHRU de Strasbourg, Strasbourg, France

Hanane, El, Haouari, Memory Resource and Research Centre of Saint-Etienne, CHU de Saint-Etienne, Saint-Etienne, France

Elise, Enderlin, Memory Resource and Research Centre of Strasbourg, CHRU de Strasbourg, Strasbourg, France

Stéphane, Epelbaum, Institute of Memory and Alzheimer's Disease (IM2A), France and Brain and Spine Institute (ICM), France UMR S 1127, Department of Neurology, AP-HP, Pitié-Salpêtrière University Hospital, Sorbonne Universities, Pierre et Marie Curie University, Paris, France

Julie, Erraud, CIC-1401 Clinical Epidemiology, CHU de Bordeaux, Bordeaux, France

Frédérique, Etcharry-Bouyx, Memory Resource and Research Centre of Angers, CHU d’Angers, Angers, France

Magali, Eyriey, Memory Resource and Research Centre of Colmar, Colmar, France

Daniel, Fagret, Memory Resource and Research Centre of Grenoble, CHU de Grenoble Alpes, Grenoble, France

Isabelle, Faillenot, Memory Resource and Research Centre of Saint-Etienne, CHU de Saint-Etienne, Saint-Etienne, France

Catherine, Faisant, Memory Resource and Research Centre of Toulouse, CHU de Toulouse, Toulouse, France

Karim, Farid, Memory Resource and Research Centre of Paris Nord, AP-HP, Paris, France

Véronique, Fasquel, Memory Resource and Research of Amiens, CHU Amiens Picardie, Amiens, France

Marion, Fatisson, Memory Resource and Research Centre of Saint-Etienne, CHU de Saint-Etienne, Saint-Etienne, France

Denis, Fédérico, Memory Resource and Research Centre of Lyon, Hospices Civils de Lyon, Lyon, France

Olivier, Felician, Memory Resource and Research Centre of Marseille, CHU de Marseille, Marseille, France

Philippe, Fernandez, Memory Resource and Research Centre of Bordeaux, CHU de Bordeaux, Bordeaux, France

Sabrina, Ferreira, Memory Resource and Research Centre of Besançon, Besançon, France

Camille, Ferté, Memory Resource and Research Centre of Lille, CHRU de Lille, Lille, France

Guillaume, Fiard, Memory Resource and Research Centre of Lyon, Hospices Civils de Lyon, Lyon, France

Florine, Fievet, Memory Resource and Research Centre of Lille, CHRU de Lille, Lille, France

Martine, Flores, Memory Resource and Research Centre of Montpellier, CHU de Montpellier, Montpellier, France

Pacôme, Fosse, Memory Resource and Research Centre of Angers, CHU d’Angers, Angers, France

Alexandra, Foubert-Samier, Memory Resource and Research Centre of Bordeaux, CHU de Bordeaux, Bordeaux, France

Sandrine, Fouchet, Memory Resource and Research Centre of Bordeaux, CHU de Bordeaux, Bordeaux, France

Marjolaine, Fourcade, Memory Resource and Research Centre of Montpellier, CHU de Montpellier, Montpellier, France

Isabelle, Franck, Memory Resource and Research Centre of Strasbourg, CHRU de Strasbourg, Strasbourg, France

Monique, Galitzky, Memory Resource and Research Centre of Toulouse, CHU de Toulouse, Toulouse, France

Céline, Gallazzini-Crepin, Memory Resource and Research Centre of Grenoble, CHU de Grenoble Alpes, Grenoble, France

Radka, Gantcheva, Memory Resource and Research Centre of Marseille, CHU de Marseille, Marseille, France

Laurence, Garbarg-Chenon, Memory Clinic of Avicenne, Hôpital Avicenne, Bobigny, France

Patrick, Gelé, Memory Resource and Research Centre of Lille, CHRU de Lille, Lille, France

Emmanuel, Gerardin, Memory Resource and Research Centre of Rouen, CHU de Rouen, Rouen, France

Pascale, Gerardin, Memory Resource and Research Centre of Nancy, CHU de Nancy, Nancy, France

Loïc, Gerlier, Memory Resource and Research Centre of Bordeaux, CHU de Bordeaux, Bordeaux, France

Claire, Gervais, Memory Resource and Research Centre of Nice, CHU de Nice, Nice, France

Jean-Claude, Getenet, Memory Resource and Research Centre of Saint-Etienne, CHU de Saint-Etienne, Saint-Etienne, France

Cindy, Giaume, Memory Resource and Research Centre of Nice, CHU de Nice, Nice, France

Carole, Girard, Memory Resource and Research Centre of Rouen, CHU de Rouen, Rouen, France

Nadine, Girard, Memory Resource and Research Centre of Marseille, CHU de Marseille, Marseille, France

Béatrice, Giroz, Memory Resource and Research Centre of Strasbourg, CHRU de Strasbourg, Strasbourg, France

Chantal, Girtanner, Memory Resource and Research Centre of Saint-Etienne, CHU de Saint-Etienne, Saint-Etienne, France

Valérie, Gissot, Memory Resource and Research Centre of Tours, CHRU de Tours, Tours, France

Blandine, Giusti, Memory Resource and Research Centre of Lyon, Hospices Civils de Lyon, Lyon, France

Patrick, Gouel, Memory Resource and Research Centre of Rouen, CHU de Rouen, Rouen, France

Natalina, Gour, Memory Resource and Research Centre of Marseille, CHU de Marseille, Marseille, France

Anne-Sophie, Gourgues, Memory Resource and Research Centre of Poitiers, CHU de Poitiers, Poitiers, France

Caroline, Grangeon, Memory Resource and Research Centre of Nice, CHU de Nice, Nice, France

Caroline, Grasselli-Monboisse, Memory Resource and Research Centre of Montpellier, CHU de Montpellier, Montpellier, France

Hélène, Gros-Dagnac, Memory Resource and Research Centre of Toulouse, CHU de Toulouse, Toulouse, France

Daniel, Grucker, Memory Resource and Research Centre of Strasbourg, CHRU de Strasbourg, Strasbourg, France

Eric, Guedj, Memory Resource and Research Centre of Marseille, CHU de Marseille, Marseille, France

Claude, Gueriot, Memory Resource and Research Centre of Marseille, CHU de Marseille, Marseille, France

Blandine, Guignard, Memory Resource and Research Centre of Strasbourg, CHRU de Strasbourg, Strasbourg, France

Yves, Guilhermet, Memory Resource and Research Centre of Lyon, Hospices Civils de Lyon, Lyon, France

Rémy, Guillevin, Memory Resource and Research Centre of Poitiers, CHU de Poitiers, Poitiers, France

Anne, Guyard, Memory Resource and Research Centre of Angers, CHU d’Angers, Angers, France

Jacques, Guyard, Memory Resource and Research Centre of Angers, CHU d’Angers, Angers, France

Lilia, Habbessi, Memory Resource and Research Centre of Lyon, Hospices Civils de Lyon, Lyon, France

Sophie, Haffen, Memory Resource and Research Centre of Besançon, Besançon, France

Sarah, Hammami, Memory Clinic of Avicenne, Hôpital Avicenne, Bobigny, France

Didier, Hannequin, Memory Resource and Research Centre of Rouen, CHU de Rouen, Rouen, France

Véronique, Hannier, Memory Resource and Research Centre of Rouen, CHU de Rouen, Rouen, France

Anne-Marie, Hanser, Memory Resource and Research Centre of Colmar, Colmar, France

Saoussen, Haouas, Memory Resource and Research Centre of Paris Broca, AP-HP, Paris, France

Anaïs, Heurtebise, Memory Resource and Research Centre of Montpellier, CHU de Montpellier, Montpellier, France

Sophie, Hierry, Memory Resource and Research Centre of Colmar, Colmar, France

Anne, Hitzel, Memory Resource and Research Centre of Toulouse, CHU de Toulouse, Toulouse, France

Claude, Hossein-Foucher, Memory Resource and Research Centre of Lille, CHRU de Lille, Lille, France

Fabrice, Hubele, Memory Resource and Research Centre of Strasbourg, CHRU de Strasbourg, Strasbourg, France

Sabrina, Iannuzzi, Memory Resource and Research Centre of Grenoble, CHU de Grenoble Alpes, Grenoble, France

Danielle, Ibarrola, Memory Resource and Research Centre of Lyon, Hospices Civils de Lyon, Lyon, France

Sandrine, Indart, Memory Resource and Research Centre of Paris Nord, AP-HP, Paris, France

Agnès, Jacquin-Piques, Memory Resource and Research Centre of Dijon, CHU Dijon Bourgogne, Dijon, France

Sophie, Jaeger, Memory Resource and Research Centre of Colmar, Colmar, France

Séverine, Jallier, Coordinating Centre, CIC-1401 Clinical Epidemiology, Bordeaux, France

Betty, Jean, Memory Resource and Research Centre of Clermont-Ferrand, CHU de Clermont-Ferrand, Clermont-Ferrand, France

Joanne, Jenn, Memory Resource and Research Centre of Bordeaux, CHU de Bordeaux, Bordeaux, France

Laure, Joly, Memory Resource and Research Centre of Nancy, CHU de Nancy, Nancy, France

Thérèse, Jonveaux, Memory Resource and Research Centre of Nancy, CHU de Nancy, Nancy, France

Séverine, Jourdain, Memory Resource and Research Centre of Rouen, CHU de Rouen, Rouen, France

Adrien, Julian, Memory Resource and Research Centre of Poitiers, CHU de Poitiers, Poitiers, France

Barbara, Jung, Memory Resource and Research Centre of Strasbourg, CHRU de Strasbourg, Strasbourg, France

Alexandra, Juphard, Memory Resource and Research Centre of Grenoble, CHU de Grenoble Alpes, Grenoble, France

Nora, Karaoun, Memory Resource and Research Centre of Paris Nord, AP-HP, Paris, France

Anisse, Karoun, CIC-1401 Clinical Epidemiology, CHU de Bordeaux, Bordeaux, France

Aurélie, Kas, Laboratoire d'Imagerie Biomédicale, Sorbonne Universités, UPMC Univ Paris 06, Inserm U 1146, CNRS UMR 7371, F-75006 Paris, France NeuroSpin, I2BM, Commissariat à l'Energie Atomique, France

Anna, Kearney-Schwartz, Memory Resource and Research Centre of Nancy, CHU de Nancy, Nancy, France

Sandrine, Keignart, Memory Resource and Research Centre of Grenoble, CHU de Grenoble Alpes, Grenoble, France

Antony, Kelly, Memory Resource and Research Centre of Clermont-Ferrand, CHU de Clermont-Ferrand, Clermont-Ferrand, France

Anne, Klebaur, Memory Resource and Research Centre of Colmar, Colmar, France

Catherine, Kleitz, Memory Resource and Research Centre of Strasbourg, CHRU de Strasbourg, Strasbourg, France

Lejla, Koric, Memory Resource and Research Centre of Marseille, CHU de Marseille, Marseille, France

Alexandre, Krainik, Memory Resource and Research Centre of Grenoble, CHU de Grenoble Alpes, Grenoble, France

Stéphane, Kremer, Memory Resource and Research Centre of Strasbourg, CHRU de Strasbourg, Strasbourg, France

Florian, Labourée, Memory Resource and Research Centre of Paris Broca, AP-HP, Paris, France

Franck, Lacoeuille, Memory Resource and Research Centre of Angers, CHU d’Angers, Angers, France

Valérie, Lafont, Memory Resource and Research Centre of Nice, CHU de Nice, Nice, France

Marie-Claude, Lagneau, Memory Resource and Research Centre of Dijon, CHU Dijon Bourgogne, Dijon, France

Sophie, Lagouarde, Memory Resource and Research Centre of Bordeaux, CHU de Bordeaux, Bordeaux, France

Francoise, Lala, Memory Resource and Research Centre of Toulouse, CHU de Toulouse, Toulouse, France

Frédéric, Lamare, Memory Resource and Research Centre of Bordeaux, CHU de Bordeaux, Bordeaux, France

Sophie, Lamarque, CIC-1401 Clinical Epidemiology, CHU de Bordeaux, Bordeaux, France

Franck, Lamberton, Memory Resource and Research Centre of Lyon, Hospices Civils de Lyon, Lyon, France

Chantal, Lamy, Memory Resource and Research of Amiens, CHU Amiens Picardie, Amiens, France

Pauline, Lapalus, Memory Resource and Research Centre of Paris Nord, AP-HP, Paris, France

Jean-Louis, Laplanche, Memory Resource and Research Centre of Paris Nord, AP-HP, Paris, France

Delphine, Lassus-Sangosse, Memory Resource and Research Centre of Grenoble, CHU de Grenoble Alpes, Grenoble, France

Caroline, Latger-Florence, Memory Resource and Research Centre of Marseille, CHU de Marseille, Marseille, France

Cyrille, Launay, Memory Resource and Research Centre of Angers, CHU d’Angers, Angers, France

Caroline, Laurent, Memory Resource and Research Centre of Lyon, Hospices Civils de Lyon, Lyon, France

Mathilde, Laye, Memory Resource and Research Centre of Nice, CHU de Nice, Nice, France

Didier, Le, Bars, Memory Resource and Research Centre of Lyon, Hospices Civils de Lyon, Lyon, France

Séverine, Le, Dily, Memory Resource and Research Centre of Nantes, CHU de Nantes, Nantes, France

Liliane, Le, Guay, Memory Resource and Research Centre of Strasbourg, CHRU de Strasbourg, Strasbourg, France

Lisa, Le, Scouarnec, CIC-1401 Clinical Epidemiology, CHU de Bordeaux, Bordeaux, France

Isabelle, Le, Taillandier, de, Gabory, Memory Resource and Research Centre of Bordeaux, CHU de Bordeaux, Bordeaux, France

Emmanuelle, Lebars, Memory Resource and Research Centre of Montpellier, CHU de Montpellier, Montpellier, France

Cécile, Lebrun-Givois, Memory Resource and Research Centre of Saint-Etienne, CHU de Saint-Etienne, Saint-Etienne, France

Eugénie, Leclerc, Memory Clinic of Avicenne, Hôpital Avicenne, Bobigny, France

Jihyun, Lee, Roy, Memory Resource and Research Centre of Nice, CHU de Nice, Nice, France

Jean-François, Legrand, Memory Resource and Research Centre of Lille, CHRU de Lille, Lille, France

Stéphane, Lehericy, Institute of Memory and Alzheimer's Disease (IM2A), France and Brain and Spine Institute (ICM), France UMR S 1127, Department of Neurology, AP-HP, Pitié-Salpêtrière University Hospital, Sorbonne Universities, Pierre et Marie Curie University, Paris, France

Sylvain, Lehmann, Memory Resource and Research Centre of Montpellier, CHU de Montpellier, Montpellier, France

Mathieu, Leininger, Memory Resource and Research Centre of Nancy, CHU de Nancy, Nancy, France

Justine, Lemaire, Memory Resource and Research Centre of Nice, CHU de Nice, Nice, France

Hermine, Lenoir, Memory Resource and Research Centre of Paris Broca, AP-HP, Paris, France

Marylin, Leny, Memory Resource and Research Centre of Paris Nord, AP-HP, Paris, France

Elsa, Leone, Memory Resource and Research Centre of Nice, CHU de Nice, Nice, France

Mélanie, Leroy, Memory Resource and Research Centre of Lille, CHRU de Lille, Lille, France

Mylène, Lesage, Memory Resource and Research Centre of Strasbourg, CHRU de Strasbourg, Strasbourg, France

Marcel, Levy, Institute of Memory and Alzheimer's Disease (IM2A), France and Brain and Spine Institute (ICM), France UMR S 1127, Department of Neurology, AP-HP, Pitié-Salpêtrière University Hospital, Sorbonne Universities, Pierre et Marie Curie University, Paris, France

Stéphanie, Libercier, Memory Resource and Research Centre of Colmar, Colmar, France

Julie, Lidier, CIC-1401 Clinical Epidemiology, CHU de Bordeaux, Bordeaux, France

Nadine, Longato, Memory Resource and Research Centre of Strasbourg, CHRU de Strasbourg, Strasbourg, France

Paulo, Loureiro, de, Sousa, Memory Resource and Research Centre of Strasbourg, CHRU de Strasbourg, Strasbourg, France

Marie, Luce, Royère, Memory Resource and Research Centre of Marseille, CHU de Marseille, Marseille, France

Juliette, Ly, Institute of Memory and Alzheimer's Disease (IM2A), France and Brain and Spine Institute (ICM), France UMR S 1127, Department of Neurology, AP-HP, Pitié-Salpêtrière University Hospital, Sorbonne Universities, Pierre et Marie Curie University, Paris, France

Marie-Anne, Mackowiak-Cordoliani, Memory Resource and Research Centre of Lille, CHRU de Lille, Lille, France

Eloi, Magnin, Memory Resource and Research Centre of Besançon, Besançon, France

Serge, Maia, Memory Resource and Research Centre of Tours, CHRU de Tours, Tours, France

Didier, Maillet, Memory Clinic of Avicenne, Hôpital Avicenne, Bobigny, France

Zaza, Makaroff, Memory Resource and Research Centre of Lyon, Hospices Civils de Lyon, Lyon, France

Oldès, Mansour, Memory Resource and Research Centre of Lyon, Hospices Civils de Lyon, Lyon, France

Athina, Marantidou, Memory Clinic of Avicenne, Hôpital Avicenne, Bobigny, France

Isabelle, Marcet, Memory Resource and Research Centre of Bordeaux, CHU de Bordeaux, Bordeaux, France

Olivier, Marcy, CIC-1401 Clinical Epidemiology, CHU de Bordeaux, Bordeaux, France

Cécilia, Marelli, Memory Resource and Research Centre of Montpellier, CHU de Montpellier, Montpellier, France

Sophie, Marilier, Memory Resource and Research Centre of Dijon, CHU Dijon Bourgogne, Dijon, France

Fanny, Marmet, Memory Resource and Research Centre of Nice, CHU de Nice, Nice, France

Laurent, Marquine, Memory Resource and Research Centre of Toulouse, CHU de Toulouse, Toulouse, France

Corinne, Marrer, Memory Resource and Research Centre of Strasbourg, CHRU de Strasbourg, Strasbourg, France

Idalie, Martin, Memory Resource and Research Centre of Lyon, Hospices Civils de Lyon, Lyon, France

Sandrine, Martin, Memory Resource and Research Centre of Montpellier, CHU de Montpellier, Montpellier, France

Olivier, Martinaud, Memory Resource and Research Centre of Rouen, CHU de Rouen, Rouen, France

Catherine, Martin-Hunyadi, Memory Resource and Research Centre of Strasbourg, CHRU de Strasbourg, Strasbourg, France

Isabelle, Mathieu, Memory Resource and Research Centre of Colmar, Colmar, France

Fabien, Maurel, Memory Resource and Research Centre of Nice, CHU de Nice, Nice, France

Sylvie, Maymon, Memory Resource and Research Centre of Colmar, Colmar, France

Joachim, Mazère, Memory Resource and Research Centre of Bordeaux, CHU de Bordeaux, Bordeaux, France

Aïcha, Medjoul, Memory Clinic of Avicenne, Hôpital Avicenne, Bobigny, France

Isabelle, Meiss, Memory Resource and Research Centre of Strasbourg, CHRU de Strasbourg, Strasbourg, France

Aurélie, Méozoone, Memory Resource and Research Centre of Paris Nord, AP-HP, Paris, France

Isabelle, Merlet, Memory Resource and Research Centre of Poitiers, CHU de Poitiers, Poitiers, France

Catherine, Mertz, Memory Resource and Research Centre of Besançon, Besançon, France

Danielle, Mestas, Memory Resource and Research Centre of Clermont-Ferrand, CHU de Clermont-Ferrand, Clermont-Ferrand, France

Catherine, Metzger, Memory Resource and Research Centre of Strasbourg, CHRU de Strasbourg, Strasbourg, France

Sabine, Meurrens, Memory Resource and Research Centre of Lille, CHRU de Lille, Lille, France

Marc-Etienne, Meyer, Memory Resource and Research of Amiens, CHU Amiens Picardie, Amiens, France

Jean-Marc, Michel, Memory Resource and Research Centre of Colmar, Colmar, France

Agnès, Michon, Institute of Memory and Alzheimer's Disease (IM2A), France and Brain and Spine Institute (ICM), France UMR S 1127, Department of Neurology, AP-HP, Pitié-Salpêtrière University Hospital, Sorbonne Universities, Pierre et Marie Curie University, Paris, France

Isabelle, Migeon-Duballet, Memory Resource and Research Centre of Poitiers, CHU de Poitiers, Poitiers, France

Carole, Miguet-Alfonsi, Memory Resource and Research Centre of Besançon, Besançon, France

Karl, Mondon, Memory Resource and Research Centre of Tours, CHRU de Tours, Tours, France

Laëtitia, Monjoin, Memory Resource and Research Centre of Strasbourg, CHRU de Strasbourg, Strasbourg, France

Pascale, Morel, Memory Resource and Research Centre of Colmar, Colmar, France

Sébastien, Moreno, Memory Resource and Research Centre of Nice, CHU de Nice, Nice, France

Clément, Morgat, Memory Resource and Research Centre of Bordeaux, CHU de Bordeaux, Bordeaux, France

Charline, Morillon, Memory Resource and Research Centre of Tours, CHRU de Tours, Tours, France

Chrystèle, Mosca, Memory Resource and Research Centre of Grenoble, CHU de Grenoble Alpes, Grenoble, France

Véronique, Moullart, Memory Resource and Research of Amiens, CHU Amiens Picardie, Amiens, France

Christian, Moussard, Memory Resource and Research Centre of Besançon, Besançon, France

Aurélie, Mouton, Memory Resource and Research Centre of Nice, CHU de Nice, Nice, France

Izzie, Jacques, Namer, Memory Resource and Research Centre of Strasbourg, CHRU de Strasbourg, Strasbourg, France

Jungalee, Navichka, Laboratoire d'Imagerie Biomédicale, Sorbonne Universités, UPMC Univ Paris 06, Inserm U 1146, CNRS UMR 7371, F-75006 Paris, France NeuroSpin, I2BM, Commissariat à l'Energie Atomique, France

Sophie, Navucet, Memory Resource and Research Centre of Montpellier, CHU de Montpellier, Montpellier, France

Raymond, Nelly, Memory Resource and Research Centre of Lyon, Hospices Civils de Lyon, Lyon, France

Thierry, Nicolas, Memory Resource and Research Centre of Besançon, Besançon, France

Georges, Niewiadomski, Memory Resource and Research Centre of Nice, CHU de Nice, Nice, France

Guillaume, Nivaggoni, Memory Resource and Research Centre of Nice, CHU de Nice, Nice, France

Marie, Noblet, Memory Resource and Research Centre of Strasbourg, CHRU de Strasbourg, Strasbourg, France

Nicolas, Noiret, Memory Resource and Research Centre of Besançon, Besançon, France

Fati, Nourhashemi, Memory Resource and Research Centre of Toulouse, CHU de Toulouse, Toulouse, France

Francis, Nyasse, Institute of Memory and Alzheimer's Disease (IM2A), France and Brain and Spine Institute (ICM), France UMR S 1127, Department of Neurology, AP-HP, Pitié-Salpêtrière University Hospital, Sorbonne Universities, Pierre et Marie Curie University, Paris, France

Estelle, Occelli, Memory Resource and Research Centre of Nice, CHU de Nice, Nice, France

Hélène, Oesterle, Memory Resource and Research Centre of Colmar, Colmar, France

Justine, Oosterlinck, Memory Resource and Research Centre of Lille, CHRU de Lille, Lille, France

Claudie, Ornon, Memory Resource and Research Centre of Poitiers, CHU de Poitiers, Poitiers, France

Galdric, Orvoen, Memory Resource and Research Centre of Paris Broca, AP-HP, Paris, France

Pierre, Jean, Ousset, Memory Resource and Research Centre of Toulouse, CHU de Toulouse, Toulouse, France

Anne, Pachart, Memory Resource and Research Centre of Colmar, Colmar, France

Florian, Palabaud, Memory Resource and Research Centre of Saint-Etienne, CHU de Saint-Etienne, Saint-Etienne, France

Juliette, Palisson, Memory Clinic of Avicenne, Hôpital Avicenne, Bobigny, France

Amandine, Pallardy, Memory Resource and Research Centre of Nantes, CHU de Nantes, Nantes, France

Sylvie, Papacatzis, Memory Resource and Research Centre of Grenoble, CHU de Grenoble Alpes, Grenoble, France

Claire, Paquet, Memory Resource and Research Centre of Paris Nord, AP-HP, Paris, France

Pierre-Yves, Pare, Memory Resource and Research Centre of Angers, CHU d’Angers, Angers, France

Guillaume, Pariscoat, Memory Resource and Research Centre of Montpellier, CHU de Montpellier, Montpellier, France

Anne, Pasco, Memory Resource and Research Centre of Angers, CHU d’Angers, Angers, France

Pierre, Payoux, Memory Resource and Research Centre of Toulouse, CHU de Toulouse, Toulouse, France

Cécile, Pays, Memory Resource and Research Centre of Montpellier, CHU de Montpellier, Montpellier, France

Julie, Pelat, Memory Resource and Research Centre of Marseille, CHU de Marseille, Marseille, France

Katell, Peoch, Phar, Memory Resource and Research Centre of Paris Nord, AP-HP, Paris, France

Rémy, Perdrisot, Memory Resource and Research Centre of Poitiers, CHU de Poitiers, Poitiers, France

Raphaël, Pereira, Memory Resource and Research Centre of Lyon, Hospices Civils de Lyon, Lyon, France

Bertille, Perin, Memory Resource and Research of Amiens, CHU Amiens Picardie, Amiens, France

Christine, Perret-Guillaume, Memory Resource and Research Centre of Nancy, CHU de Nancy, Nancy, France

Sophie, Pérusat, Coordinating Centre, CIC-1401 Clinical Epidemiology, Clinical Project Manager, France

Yolande, Petegnief, Memory Resource and Research Centre of Besançon, Besançon, France

Grégory, Petyt, Memory Resource and Research Centre of Lille, CHRU de Lille, Lille, France

Lorène, Philibert, Memory Resource and Research Centre of Nice, CHU de Nice, Nice, France

Nathalie, Philippi, Memory Resource and Research Centre of Strasbourg, CHRU de Strasbourg, Strasbourg, France

Clélie, Phillipps, Memory Resource and Research Centre of Strasbourg, CHRU de Strasbourg, Strasbourg, France

Julie, Piano, Memory Resource and Research Centre of Nice, CHU de Nice, Nice, France

Michèle, Pierre, Memory Resource and Research Centre of Toulouse, CHU de Toulouse, Toulouse, France

Johan, Pietras, Memory Resource and Research Centre of Grenoble, CHU de Grenoble Alpes, Grenoble, France

Mélanie, Pigot, Memory Resource and Research Centre of Montpellier, CHU de Montpellier, Montpellier, France

Fanny, Pineau, Memory Resource and Research Centre of Colmar, Colmar, France

Geneviève, Pinganaud, Memory Resource and Research Centre of Bordeaux, CHU de Bordeaux, Bordeaux, France

Pierre, Pitet, Memory Resource and Research Centre of Grenoble, CHU de Grenoble Alpes, Grenoble, France

Matthieu, Plichart, Memory Resource and Research Centre of Paris Broca, AP-HP, Paris, France

Catherine, Poisson, Institute of Memory and Alzheimer's Disease (IM2A), France and Brain and Spine Institute (ICM), France UMR S 1127, Department of Neurology, AP-HP, Pitié-Salpêtrière University Hospital, Sorbonne Universities, Pierre et Marie Curie University, Paris, France

Elodie, Pongan, Memory Resource and Research Centre of Lyon, Hospices Civils de Lyon, Lyon, France

Gabriel, Pop, Memory Clinic of Avicenne, Hôpital Avicenne, Bobigny, France

Dorothée, Pouliquen, Memory Resource and Research Centre of Rouen, CHU de Rouen, Rouen, France

Cyril, Poupon, Institute of Memory and Alzheimer's Disease (IM2A), France and Brain and Spine Institute (ICM), France UMR S 1127, Department of Neurology, AP-HP, Pitié-Salpêtrière University Hospital, Sorbonne Universities, Pierre et Marie Curie University, Paris, France

Stéphane, Pouponneau, Memory Resource and Research Centre of Tours, CHRU de Tours, Tours, France

Bruno, Pozetto, Memory Resource and Research Centre of Saint-Etienne, CHU de Saint-Etienne, Saint-Etienne, France

Sophie, Pradier, Memory Resource and Research Centre of Bordeaux, CHU de Bordeaux, Bordeaux, France

Thierry, Prangère, Memory Resource and Research Centre of Lille, CHRU de Lille, Lille, France

Magali, Prévot, Memory Resource and Research Centre of Paris Nord, AP-HP, Paris, France

Evelyne, Provost, Memory Resource and Research Centre of Saint-Etienne, CHU de Saint-Etienne, Saint-Etienne, France

Michèle, Puel, Memory Resource and Research Centre of Toulouse, CHU de Toulouse, Toulouse, France

Mathieu, Queneau, Memory Resource and Research Centre of Paris Nord, AP-HP, Paris, France

Muriel, Quillard-Muraine, Memory Resource and Research Centre of Rouen, CHU de Rouen, Rouen, France

Valérie, Quipourt, Memory Resource and Research Centre of Dijon, CHU Dijon Bourgogne, Dijon, France

Chloé, Rachez, Memory Resource and Research Centre of Clermont-Ferrand, CHU de Clermont-Ferrand, Clermont-Ferrand, France

Aline, Rahnema, Memory Resource and Research Centre of Nancy, CHU de Nancy, Nancy, France

Muriel, Rainfray, Memory Resource and Research Centre of Bordeaux, CHU de Bordeaux, Bordeaux, France

Nadine, Raoux, Memory Resource and Research Centre of Bordeaux, CHU de Bordeaux, Bordeaux, France

Anatta, Razafimanantsoa, Memory Clinic of Avicenne, Hôpital Avicenne, Bobigny, France

Micheline, Razzouk-Cadet, Memory Resource and Research Centre of Nice, CHU de Nice, Nice, France

Maria, Rego-Lopes, Memory Resource and Research Centre of Paris Broca, AP-HP, Paris, France

Solveig, Relland, Memory Resource and Research Centre of Lyon, Hospices Civils de Lyon, Lyon, France

Marie, Revillon, Institute of Memory and Alzheimer's Disease (IM2A, France and Brain and Spine Institute (ICM, France UMR S 1127, Department of Neurology, AP-HP, Pitié-Salpêtrière University Hospital, Sorbonne Universities, Pierre et Marie Curie University, France

Sylvie, Richard, Memory Resource and Research Centre of Lyon, Hospices Civils de Lyon, Lyon, France

Virginie, Richard, Coordinating Centre, CIC-1401 Clinical Epidemiology, Data Manager, France

Eliane, Riera, Memory Resource and Research Centre of Colmar, Colmar, France

Anne-Sophie, Rigaud, Memory Resource and Research Centre of Paris Broca, AP-HP, Paris, France

Marie-Claire, Riocreux, Memory Resource and Research Centre of Saint-Etienne, CHU de Saint-Etienne, Saint-Etienne, France

Philippe, Robert, Memory Resource and Research Centre of Nice, CHU de Nice, Nice, France

Hélène, Robin-Ismer, Memory Resource and Research Centre of Strasbourg, CHRU de Strasbourg, Strasbourg, France

Laëtitia, Rocher, Memory Resource and Research Centre of Nantes, CHU de Nantes, Nantes, France

Fabienne, Rochette, Memory Resource and Research Centre of Grenoble, CHU de Grenoble Alpes, Grenoble, France

Mathieu, Rodallec, Memory Resource and Research Centre of Paris Nord, AP-HP, Paris, France

Yves, Rolland, Memory Resource and Research Centre of Toulouse, CHU de Toulouse, Toulouse, France

Adeline, Rollin-Sillaire, Memory Resource and Research Centre of Lille, CHRU de Lille, Lille, France

Fabien, Rondepierre, Memory Resource and Research Centre of Clermont-Ferrand, CHU de Clermont-Ferrand, Clermont-Ferrand, France

Stéphanie, Roseng, Coordinating Centre, CIC-1401 Clinical Epidemiology, Clinical Research Associate, France

Mélanie, Rossitto, Memory Resource and Research Centre of Nancy, CHU de Nancy, Nancy, France

Caroline, Roubaud, Memory Resource and Research Centre of Lyon, Hospices Civils de Lyon, Lyon, France

Isabelle, Rouch, Memory Resource and Research Centre of Lyon, Hospices Civils de Lyon, Lyon, France

Olivier, Roulant, Memory Resource and Research Centre of Toulouse, CHU de Toulouse, Toulouse, France

Martine, Roussel, Memory Resource and Research of Amiens, CHU Amiens Picardie, Amiens, France

Annie, Routier, Memory Resource and Research of Amiens, CHU Amiens Picardie, Amiens, France

Julie, Roux, Memory Resource and Research Centre of Grenoble, CHU de Grenoble Alpes, Grenoble, France

Perrine, Roy, Institute of Memory and Alzheimer's Disease (IM2A, France and Brain and Spine Institute (ICM, France UMR S 1127, Department of Neurology, AP-HP, Pitié-Salpêtrière University Hospital, Sorbonne Universities, Pierre et Marie Curie University, France

Séverine, Roy, Memory Resource and Research Centre of Lyon, Hospices Civils de Lyon, Lyon, France

Ilham, Ryff, Memory Resource and Research Centre of Besançon, Besançon, France

Guillaume, Sacco, Memory Resource and Research Centre of Nice, CHU de Nice, Nice, France

Djamel, Saidi, Memory Resource and Research Centre of Bordeaux, CHU de Bordeaux, Bordeaux, France

Anne-Sophie, Salabert, Memory Resource and Research Centre of Toulouse, CHU de Toulouse, Toulouse, France

François, Salmon, Memory Resource and Research Centre of Poitiers, CHU de Poitiers, Poitiers, France

Maria-Joao, Santiago-Ribeiro, Memory Resource and Research Centre of Tours, CHRU de Tours, Tours, France

Joëlle, Sapin, Memory Resource and Research Centre of Bordeaux, CHU de Bordeaux, Bordeaux, France

Nadine, Sapin, Memory Resource and Research Centre of Nice, CHU de Nice, Nice, France

Alain, Sarciron, Memory Resource and Research Centre of Lyon, Hospices Civils de Lyon, Lyon, France

Nathalie, Sastres, Memory Resource and Research Centre of Toulouse, CHU de Toulouse, Toulouse, France

Amandine, Saubion, Memory Resource and Research Centre of Toulouse, CHU de Toulouse, Toulouse, France

Mathilde, Sauvée, Memory Resource and Research Centre of Grenoble, CHU de Grenoble Alpes, Grenoble, France

Sophie, Schahl, Memory Resource and Research Centre of Colmar, Colmar, France

Christian, Scheiber, Memory Resource and Research Centre of Lyon, Hospices Civils de Lyon, Lyon, France

Aude, Schlecht, Memory Resource and Research Centre of Colmar, Colmar, France

Anne-Marie, Schneider, Memory Resource and Research Centre of Strasbourg, CHRU de Strasbourg, Strasbourg, France

Floraly, Sejalon, Memory Clinic of Avicenne, Hôpital Avicenne, Bobigny, France

Christiane, Sergent, Memory Resource and Research Centre of Bordeaux, CHU de Bordeaux, Bordeaux, France

Amélie, Serra, Memory Resource and Research Centre of Grenoble, CHU de Grenoble Alpes, Grenoble, France

Marie-Laure, Seux, Memory Resource and Research Centre of Paris Broca, AP-HP, Paris, France

Romain, Simon, Memory Resource and Research Centre of Angers, CHU d’Angers, Angers, France

Valérie, Simon, Institute of Memory and Alzheimer's Disease (IM2A, France and Brain and Spine Institute (ICM, France UMR S 1127, Department of Neurology, AP-HP, Pitié-Salpêtrière University Hospital, Sorbonne Universities, Pierre et Marie Curie University, France

Rémi, Sitta, Coordinating Centre, CIC-1401 Clinical Epidemiology, France

Hélène, Sordet-Guépet, Memory Resource and Research Centre of Dijon, CHU Dijon Bourgogne, Dijon, France

Violette, Sorel, Memory Resource and Research Centre of Lille, CHRU de Lille, Lille, France

Maria, Eugenia, Soto, Memory Resource and Research Centre of Toulouse, CHU de Toulouse, Toulouse, France

Noui, Souakri, Memory Resource and Research Centre of Bordeaux, CHU de Bordeaux, Bordeaux, France

Jacqueline, Suquet, Memory Resource and Research Centre of Montpellier, CHU de Montpellier, Montpellier, France

Géraldine, Sylvestre, Memory Resource and Research Centre of Besançon, Besançon, France

Mathieu, Tafani, Memory Resource and Research Centre of Toulouse, CHU de Toulouse, Toulouse, France

Stéphanie, Taglang, Memory Resource and Research Centre of Colmar, Colmar, France

Jean-Yves, Tanguy, Memory Resource and Research Centre of Angers, CHU d’Angers, Angers, France

Lorraine, Templier, Memory Resource and Research Centre of Paris Nord, AP-HP, Paris, France

Catherine, Terrat, Memory Resource and Research Centre of Saint-Etienne, CHU de Saint-Etienne, Saint-Etienne, France

Jamila, Thabet, Memory Clinic of Avicenne, Hôpital Avicenne, Bobigny, France

Claire, Thalamas, Memory Resource and Research Centre of Toulouse, CHU de Toulouse, Toulouse, France

Nathalie, Thiery, Coordinating Centre, CIC-1401 Clinical Epidemiology, France

François, Tison, Memory Resource and Research Centre of Bordeaux, CHU de Bordeaux, Bordeaux, France

Hélène, Ton, Van, Memory Clinic of Avicenne, Hôpital Avicenne, Bobigny, France

Lucie, Toulemonde, Memory Resource and Research Centre of Marseille, CHU de Marseille, Marseille, France

Virginie, Tourbier, Memory Resource and Research of Amiens, CHU Amiens Picardie, Amiens, France

Bertrand, Toussaint, Memory Resource and Research Centre of Grenoble, CHU de Grenoble Alpes, Grenoble, France

Eve, Tramoni, Memory Resource and Research Centre of Marseille, CHU de Marseille, Marseille, France

Candice, Trocmé, Memory Resource and Research Centre of Grenoble, CHU de Grenoble Alpes, Grenoble, France

Irène, Troprès, Memory Resource and Research Centre of Grenoble, CHU de Grenoble Alpes, Grenoble, France

Anne-Cécile, Troussière, Memory Resource and Research Centre of Lille, CHRU de Lille, Lille, France

Anne, Turazzi, Memory Resource and Research Centre of Nice, CHU de Nice, Nice, France

Renata, Ursu, Memory Clinic of Avicenne, Hôpital Avicenne, Bobigny, France

Emilie, Vaillant, Memory Resource and Research Centre of Nice, CHU de Nice, Nice, France

Nathalie, Vayssière, Memory Resource and Research Centre of Toulouse, CHU de Toulouse, Toulouse, France

Pierre, Vera, Memory Resource and Research Centre of Rouen, CHU de Rouen, Rouen, France

Olivier, Vercruysse, Memory Resource and Research Centre of Lille, CHRU de Lille, Lille, France

Antoine, Verger, Memory Resource and Research Centre of Nancy, CHU de Nancy, Nancy, France

Maximilien, Vermandel, Memory Resource and Research Centre of Lille, CHRU de Lille, Lille, France

Philippe, Viau, Memory Resource and Research Centre of Nice, CHU de Nice, Nice, France

Marie-Neige, Videau, Memory Resource and Research Centre of Bordeaux, CHU de Bordeaux, Bordeaux, France

Jean-Louis, Vincent, Memory Resource and Research Centre of Lille, CHRU de Lille, Lille, France

Vincent, Visneux, Memory Resource and Research Centre of Saint-Etienne, CHU de Saint-Etienne, Saint-Etienne, France

Isabelle, Vivier, Memory Resource and Research Centre of Bordeaux, CHU de Bordeaux, Bordeaux, France

Christelle, Vlaemynck, Memory Resource and Research Centre of Clermont-Ferrand, CHU de Clermont-Ferrand, Clermont-Ferrand, France

Natacha, Vogt, Memory Resource and Research Centre of Strasbourg, CHRU de Strasbourg, Strasbourg, France

Thierry, Voisin, Memory Resource and Research Centre of Toulouse, CHU de Toulouse, Toulouse, France

Elodie, Vulliez, Memory Resource and Research Centre of Lyon, Hospices Civils de Lyon, Lyon, France

Nathalie, Wagemann, Memory Resource and Research Centre of Nantes, CHU de Nantes, Nantes, France

Caroline, Wagner, Memory Resource and Research Centre of Strasbourg, CHRU de Strasbourg, Strasbourg, France

Aziza, Waissi-Sediq, Memory Resource and Research Centre of Lyon, Hospices Civils de Lyon, Lyon, France

Sandrine, Wannepain, Memory Resource and Research of Amiens, CHU Amiens Picardie, Amiens, France

Marie-Joséphine, Waryn, Memory Clinic of Avicenne, Hôpital Avicenne, Bobigny, France

Brigitte, Weidmann, Memory Resource and Research Centre of Colmar, Colmar, France

Emilie, Wenish, Memory Resource and Research Centre of Marseille, CHU de Marseille, Marseille, France

Léocadie, Werle, Memory Resource and Research Centre of Strasbourg, CHRU de Strasbourg, Strasbourg, France

Gabrielle, Woehrel, Memory Resource and Research Centre of Strasbourg, CHRU de Strasbourg, Strasbourg, France

Jing, Xie, Memory Resource and Research Centre of Lyon, Hospices Civils de Lyon, Lyon, France

Nathanaëlle, Yeni, Laboratoire d'Imagerie Biomédicale, Sorbonne Universités, UPMC Univ Paris 06, Inserm U 1146, CNRS UMR 7371, F-75006 Paris, France NeuroSpin, I2BM, Commissariat à l'Energie Atomique, France

Michel, Zanca, Memory Resource and Research Centre of Montpellier, CHU de Montpellier, Montpellier, France

Rupestre, Zannou, Coordinating Centre, CIC-1401 Clinical Epidemiology, Clinical Research Associate, France

Jean, Zinszner, Memory Clinic of Avicenne, Hôpital Avicenne, Bobigny, France
